# Effectiveness of Intervention Strategies to Increase Adolescents’ Physical Activity and Reduce Sedentary Time in Secondary School Settings, Including Factors Related to Implementation: A Systematic Review and Meta-Analysis

**DOI:** 10.1186/s40798-024-00688-7

**Published:** 2024-03-13

**Authors:** Ana María Contardo Ayala, Kate Parker, Emiliano Mazzoli, Natalie Lander, Nicola D. Ridgers, Anna Timperio, David R. Lubans, Gavin Abbott, Harriet Koorts, Jo Salmon

**Affiliations:** 1 Institute for Physical Activity and Nutrition (IPAN), Deakin University, Geelong, Victoria Australia; 2 Alliance for Research in Exercise, Nutrition and Activity (ARENA), Allied Health & Human Performance, University of South Australia, Adelaide, South Australia, Australia; 3 Centre for Active Living and Learning, College of Human and Social Futures, University of Newcastle, Newcastle, NSW Australia; 4 School of Exercise and Nutrition Sciences, Deakin University, Geelong, Victoria Australia; 5 School of Health and Social Development, Deakin University, Geelong, Victoria Australia; 6 Faculty of Sport and Health Sciences, University of Jyväskylä, Jyväskylä, Finland; 7 Hunter Medical Research institute, New Lambton Heights, NSW Australia

**Keywords:** Adolescents, Physical activity, Sedentary behaviour, School-based interventions, Implementation

## Abstract

**Background:**

Globally, just one in five adolescents meet physical activity guidelines and three-quarters of the school day is spent sitting. It is unclear which types of school-based interventions strategies increase physical activity and reduce sedentary time among adolescents, or how these interventions are implemented influences their effectiveness.

**Objective:**

The three aims of our systematic review were to (a) identify intervention strategies used within secondary school settings to improve students’ movement behaviours throughout school-based initiatives, delivered at or by the school; (b) determine the overall effect of the interventions (meta-analysis) on physical activity (all intensities), sedentary time, cognitive/academic, physical health and/or psychological outcomes; and (c) describe factors related to intervention implementation.

**Methods:**

Searches were conducted in MEDLINE complete, EMBASE, CINAHL, SPORTDiscus, APA PsycINFO, and ERIC in January 2023 for studies that (a) included high school-aged adolescents; (b) involved a school-based intervention to increase physical activity and/or decrease sedentary time; and (c) were published in English. Reported effects were pooled in meta-analyses where sufficient data were obtained.

**Results:**

Eighty-five articles, representing 61 interventions, met the inclusion criteria, with 23 unique intervention strategies used. Interventions that involved whole-school approaches (i.e., physical activity sessions, environmental modifications, teacher training, peer support and/or educational resources) were favourably associated with most of the outcomes. The meta-analyses showed: (a) non-significant effects for sedentary time (Standardized mean difference [SMD] = -0.02; 95%CI, -0.14, 0.11), physical activity at all intensities (light: SMD= -0.01; 95%CI, -0.08, 0.05; moderate: SMD = 0.06; 95%CI, -0.09, 0.22; vigorous: SMD = 0.08; 95%CI, -0.02, 0.18; moderate-to-vigorous: SMD = 0.05; 95%CI, -0.01, 0.12) and waist circumference (SMD = 0.09; 95%CI, -0.03, 0.21), and (b) a small statistically significant decrease in body mass index (SMD= -0.09, 95%CI -0.16, -0.0). Factors related to intervention implementation were reported in 51% of the articles.

**Conclusion:**

While some intervention approaches demonstrated promise, small or null effects were found in meta-analyses. Future school-based interventions should utilize a whole-school approach designed to increase adolescents’ activity across the day. Consistent reporting of implementation will increase understanding of how interventions are adopted, implemented and sustained.

**Registration:**

PROSPERO (CRD42020169988).

**Supplementary Information:**

The online version contains supplementary material available at 10.1186/s40798-024-00688-7.

## Key Points

- School-based interventions with adolescents have had null effects on increasing physical activity and reducing sedentary time.
- The impact on physical and psychological outcomes has been poorly reported among secondary school students.
- This review included articles with all experimental study designs, any intervention length, and that reported a variety of outcomes among adolescents in secondary schools (11–18 years old).
- Although meta-analyses showed that these interventions did not increase physical activity or reduce sedentary time, studies that quantify adiposity markers showed a small decrease in body mass index.
- The review identified that additional physical activity sessions, environmental modifications, teacher training, peer support, educational resources and/or active lesson strategies were associated with greater benefits.
- Fidelity and participant responses were the most commonly reported implementation factors, adaptation was the least commonly reported; however, factors associated with effective implementation were not a focus of the included studies.

**Supplementary Information:**

The online version contains supplementary material available at 10.1186/s40798-024-00688-7.

## Background

Adequate physical activity has a multitude of benefits for adolescents including a reduced risk of developing adverse psychological [1] and physical health conditions [2]. Moreover, physical activity is positively associated with academic outcomes, including cognitive skills (e.g., executive functioning, memory) [3], attitude (e.g., motivation, self-concept) [4], academic behaviour (e.g., on-task time, organization) [5], and academic achievement (e.g., standardized test scores) [6]. Conversely, excess sedentary time particularly recreational screen-time, during adolescence has negative implications for psychological [1] and physical health [7]. It is currently recommended that adolescents engage in at least an average of 60 min per day of moderate- to vigorous-intensity physical activity (MVPA) and limit the amount of time they spend sedentary [8].

Approximately 81% of adolescents globally do not meet physical activity guidelines [9]. Additionally, adolescents spend an average of nine hours per day sitting, which includes three hours per day engaged in sedentary screen-time [10]. Secondary (middle and high) school students can spend up to 75% of their class time sedentary; often accumulated in long, unbroken bouts of sitting [11]. Although secondary schools are typically required to provide regular physical education (PE) classes, just 36% of this time is spent in MVPA [12]. Moreover, scheduled PE time declines across secondary school years and is not always compulsory in the upper secondary or high school years [13]. There are many other opportunities for students to engage in movement behaviours (i.e., increase physical activity and/or reduce sedentary time) throughout the day, including recess and lunch breaks, and during, between and after lessons [14].

Several reviews have summarised school-based movement behaviour initiatives in the secondary school context, concluding that these have been largely ineffective (null to small positive effects) [15–23]. However, the majority have reported intervention effects on MVPA [15, 16, 18, 21], or sedentary time [23], included just PE interventions (already contributing to school-hours physical activity) [16, 19, 21], focused on older adolescents [17] or girls only [22], restricted inclusion criteria to specific intervention designs (only RCTs) [17, 18] and study lengths [15, 18], or focused on low-middle income countries [20]. Meta-analyses were performed in only five reviews [15, 16, 18, 21, 23] that described the impact of school-based interventions on physical activity (small or null effects) [16, 18, 21], and just two analyzed the intervention components used [15, 16]. None have reported the factors crucial for intervention implementation effectiveness.

To our knowledge, there is currently no synthesis of non-PE interventions delivered at or by schools to improve movement behaviours across the school day, and existing reviews have generally not reported outcomes beyond movement behaviours, such as cognitive and academic outcomes, physical health, and/or psychological outcomes. The three aims of our systematic review were to (a) identify intervention strategies used within secondary school settings to improve students’ movement behaviours throughout school-based initiatives, delivered at or by the school; (b) determine the overall effect of the interventions (meta-analysis) on physical activity (all intensities), sedentary time, cognitive/academic, physical health and/or psychological outcomes; and (c) describe factors related to intervention implementation.

## Methods

### Protocol and Registration

This review was registered with PROSPERO (CRD42020169988) and followed the Preferred Reporting Items for Systematic Reviews and Meta-Analyses Statement guidelines (PRISMA). Following the PROSPERO registration, the eligibility criteria were expanded to include strategies targeting school-related work (i.e. homework) and a third aim was added: to describe factors related to intervention implementation.

### Eligibility Criteria and Search Strategies

Eligibility criteria and search strategy were modelled on the Participants, Interventions, Comparisons, Outcomes, and Study design (PICOS) framework. Studies were included if they included: (a) Secondary/middle/high school-age adolescents (> 11 years and < 18 years); (b) Interventions delivered in the school setting (i.e., during class, recess and lunch time) and/or related to schoolwork (e.g., homework; c) Strategy/ies to increase physical activity of any intensity (i.e., light, moderate, vigorous or moderate to vigorous) and/or decrease sedentary behaviour); d) Any outcomes (for movement behaviours measures, have to report an effect on daily and/or school-based physical activity and sedentary behaviour); e) Any study design (e.g., randomized controlled trials [individual and cluster], controlled trials, pre-post studies design, quasi-experimental studies), and with any comparison (e.g., pre-post intervention comparison) or control groups (e.g., non-exposed control/comparison groups); f) written in English. Studies were excluded from this review if they included: (a) Target population was adolescents with special needs; primary (elementary), middle and secondary school studies were combined with results reported together; participants were in year 6 only (considered a primary school year in some countries); (b) Before- or after-school hours programs; (c) Intervention targeted PE lessons, before- or after-school hours, active travel, or educational programs (e.g., non-active lifestyle/health lessons) in isolation; (d) Non-experimental studies (e.g., cross-sectional and case studies); and (e) Findings were only reported in abstracts (including poster abstracts), conference proceedings, dissertations, commentaries, editorials, review articles, and letters. The search strategy is reported in Supplementary Table 1 (Search strategy used [i.e., EBSCO and EMBASE]).

### Information Sources

A systematic search was conducted using six online databases: MEDLINE complete, Embase, CINAHL, SPORTDiscus, APA PsycINFO, and ERIC. Further articles were identified via forwards and backward citation tracking of included articles and relevant systematic reviews. Peer-reviewed articles between January 2000 and January 2023 were considered for inclusion, as most school-based movement behaviour interventions have been conducted in the last ~ 20 years. Reference lists of the included articles were also screened to identify additional eligible interventions.

### Study Selection

All search results were exported into a reference manager (Endnote x9, Clarivate analytics) and duplicates were removed. Titles and abstracts were exported to Covidence (Melbourne, Australia). Two authors (KP, AMC) screened all titles and abstracts; discussed discrepancies and came to a consensus for inclusion. Both authors reviewed the full text and discussed full-text discrepancies. Any disagreements were solved in a meeting involving four authors (AMC, KP, AT, JS).

**Fig. 1:**
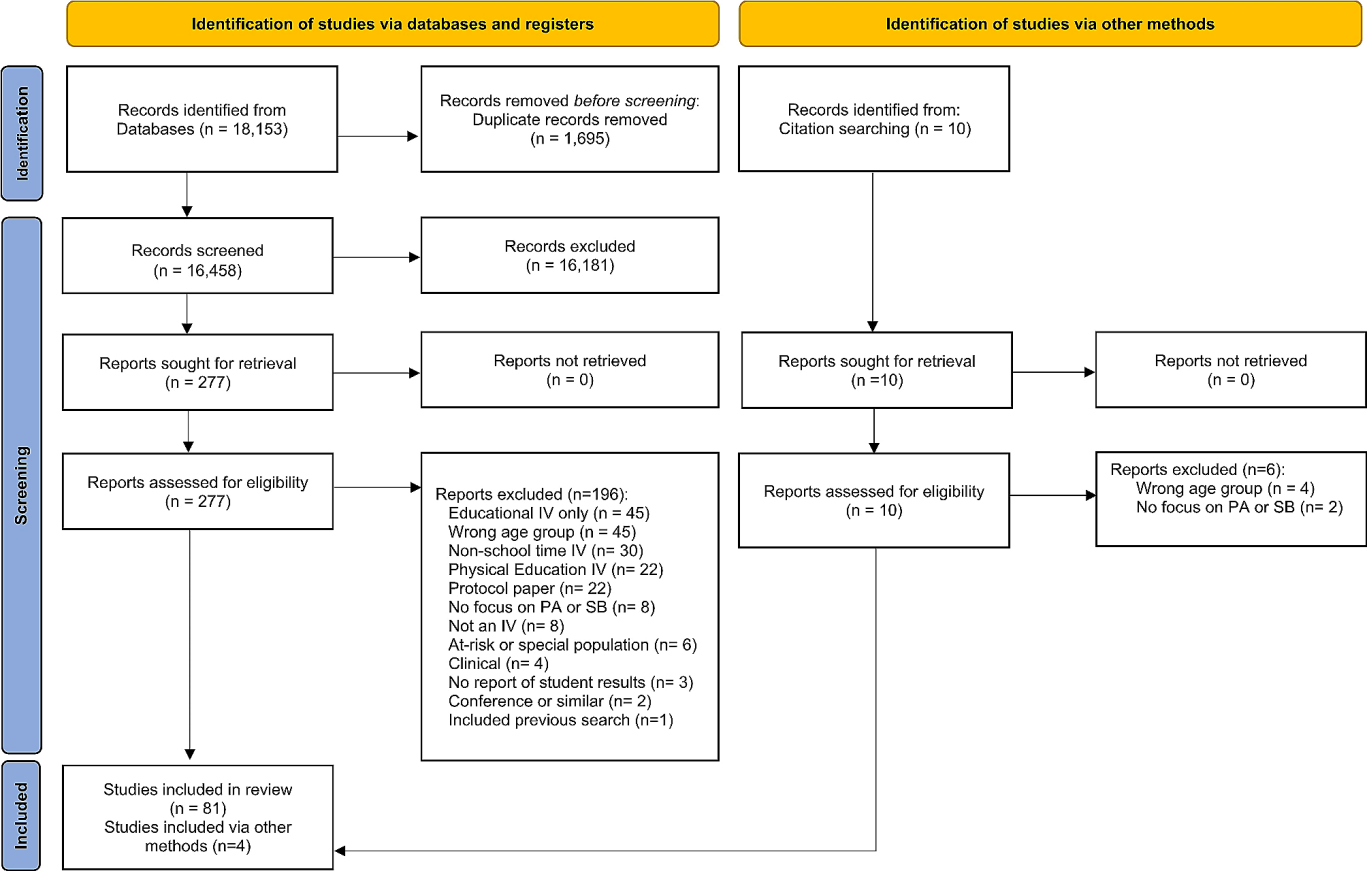
Study selection process (PRISMA − 2020 version). Legend: IV, intervention; PA, physical activity; SB, sedentary behaviour

### Data Collection Process

After identifying published articles, the authors identified whether they constituted individual studies or multiple articles from the same study. Hereafter, “articles” refers to the count of papers identified, while “studies” pertains to the number of distinct research studies/interventions represented by these articles. Data extraction was performed by two authors (AMC and KP), and all articles were cross-checked for accuracy by an additional author (EM). Data extracted and quantified included study and participant characteristics, intervention strategies and intervention effects. Intervention outcomes were classified into five categories: movement behaviours (e.g., physical activity, sedentary time, energy expenditure), cognitive and academic outcomes (e.g., working memory, on task behaviour), physical health (e.g., fitness, obesity, and musculoskeletal health), and psychological outcomes. Data extraction of factors relevant to the implementation of the programs (performed by AMC and KP; checked for accuracy by NL) was guided by the United Kingdom Medical Research Council Process Evaluation of Complex Interventions [24], which includes the following three components: *Implementation* (implementation process, fidelity, dose, adaptations, reach), *Mechanism of impact* (participants’ responses, mediators, unanticipated pathways/consequences, and *Context* (contextual factors which affect/shape intervention). Descriptive analysis was used to describe studies, participants’ characteristics and intervention components and effects on physical activity (all intensities), sedentary time, cognitive/academic, physical health, and/or psychological outcomes.

### Methodological Quality

Quality assessment screening was completed by three authors (AMC, KP, NL), in duplicate, using an adapted version of the Effective Public Health Practice Project tool for quantitative studies [25]. The three authors discussed discrepancies and came to a consensus for the scores. The components assessed were selection bias (sample representation), study design (e.g., RCT), confounders (e.g., control for baseline differences), blinding (researcher and participant), data collection methods (e.g., validity and reliability of the assessment tool used), withdrawals and dropouts (e.g., report of number of withdrawals and reason), intervention integrity (e.g., percentage of participants that received the intervention), and statistical analysis (e.g., use of intention to treat approach) [26]. Individual criteria for methodological quality scores and an overall quality score were created and rated as strong (no weak rating), moderate (one weak rating) or weak (> 1 weak rating) [25]. Inter-rater reliability for assessment of study quality between authors was moderate (Cohen’s Kappa = 0.6, *p* < 0.001).

### Statistical Analysis

Descriptive analysis (number and/or percentage) was used to summarise studies and participant characteristics, intervention strategies and the effect of these strategies on physical activity (all intensities), sedentary time, cognitive/academic, physical health and/or psychological outcomes, and factors related to intervention implementation. In addition, meta-analyses were conducted using Stata v18 when at least three articles reported intervention effects on outcomes with a comparable device (e.g., actigraphy), same outcome (e.g. daily MPA, and/or measurement scales/unit (kg/m^2^, cm), provided complete data for pre- and post- intervention measurements, and were randomised controlled trials. Self-reported movement behaviour outcomes were excluded from the meta-analyses. Consequently, meta-analyses included articles reporting on comparable: (a) device-measured movement behaviours (i.e. sedentary time, LPA, MPA, VPA, MVPA assessed during the whole day); and (b) body mass index and waist circumference (measured with standardized protocols). The sample size, mean difference, and standard error/confidence interval between intervention and control groups were entered into Microsoft® Excel and effect sizes were calculated (due to the varying units for a given outcome, standardized mean differences were calculated and used in meta-analysis). Reported immediate intervention effects were used for these meta-analyses and, in case of incomplete reporting, the corresponding authors were contacted to request additional information. For cluster randomized controlled trials, the point estimate and the 95% confidence interval intervention-reported data from their intervention effect that accounted for clustering were extracted. For cluster-randomised controlled trials, we ensured that all studies included in the meta-analyses accounted for clustered data in their statistical analyses. In cases where a study included multiple groups receiving the same or different intervention, a group combination to create a single pair-wise comparison was used [28]. A random-effects restricted maximum-likelihood model was utilized for the meta-analyses.

The Q statistic and *I*^2^ [27], and visual inspection of the forest plots, were used to examine statistical heterogeneity. *I*^2^ indicated high heterogeneity when > 75% and moderate when > 50%. Random effect meta-regression models were used to explore heterogeneity induced by the relationship between moderators (i.e., participants’ age [years], duration of the intervention [weeks], type of intervention [single-or multi-component]) and study effect sizes, when there were more than ten studies in the meta-analysis [28]. Where the meta-regression suggested the presence of a potentially important covariate, subgroup analyses were used to further investigate the data. For all meta-analysis and meta-regression models, statistical significance was set at *p* < 0.05 and effect sizes were interpreted as 0.2 small, 0.5 medium and 0.6 as large [29]. To assess potential small-study effects and publication bias for meta-analysis with at least 10 studies (i.e. MVPA, BMI, and WC) [30], funnel plots were produced and the Egger regression asymmetry test [31] was conducted. The trim-and-fill [32] computation was also used to assess the effect of publication bias on the interpretation of results.

## Results

### Characteristics of the Included Studies

In total, 85 articles (from 63 studies) fulfilled the inclusion criteria (Table 1). Of the included studies, 17 (27%) were conducted in Australia, followed by the United Kingdom (*n* = 9, 14.3%), the USA (*n* = 6, 9.5%) and other countries (*n* = 31, 49%). Study sample sizes ranged from 33 [33] to 6,476 [34] (Table 1). In total, the studies included 45,733 participants, with 50 articles (58.9%) reporting on physical activity, 31 (36.4%) on sedentary time, 35 (41.2%) on physical health, and 12 (14.1%) on psychological outcomes. Participant ages ranged from 11 to 17 years and the average age was 13.7 years (reported in 44 articles, 51.8%). The percentage of girls varied between 36% [35] and 100% [36–41]. The participants’ sex was not reported in five studies [34, 42–45]. Most of the studies (*n* = 46, 73%) used a randomised controlled trial design [33–39, 42, 44, 46–78], 15 studies (23.8%) used a quasi-experimental design [40, 79–91], one study (1.6%) used a cross-over trial design [43] and one (1.6%) a hybrid effectiveness-implementation design [92]. Intervention length ranged from a single 20-min session [52, 62] to three years [81].

**Table 1:** Characteristics of the included studies and study populations description

Authors	Design^*^	n	Sex (%)	Age (y) (Mean, range)	Length (weeks)	Outcomes^‡^	Overall methodological quality rating
Aceves-Martins et al., 2017 [55]	RCT - cluster	393	I = 50.6% B C = 47.5% B	14.6 ± 0.7, 13–16	52	PA, SED	Moderate
Ahmed et al., 2022 [95]	RCT - cluster	320	I = 65.6% B C = 51.9% B	I = 14.4 ± 1.2 C = 14.2 ± 0.9, 13–17	12	PA, SED	Weak
Altunkurek et al., 2019 [54]	RCT	132	I_1_: 42.4% B I_2_: 50% B C: 51.5% B	12–15	12	PA, WB	Weak
Amoah et al., 2021 [94]	RCT - cluster	848	51.3% G	16.9 ± 1.4 14–19	26	PA, H	Weak
Andrade et al., 2014 [70]	RCT – cluster	1234	37.6% B 62.4% G	12.8 ± 0.8	121	PA, SED, H, F	Moderate
Andrade et al., 2015 [96]						SED	Moderate
Ardic et al., 2016 [89]	Quasi - Ex	87	50.6% G	12.8 ± 0.8, 12–15	52	PA, H	Weak
Barbosa Filho et al., 2016 [63]	RCT - cluster	1085	I = 51.8% B C = 51.2% B	11–17	17	PA	Moderate
Barbosa Filho et al., 2017 [125]						PA, H	Moderate
Barbosa Filho et al., 2019 [97]						PA	Moderate
Bandeira et al., 2020 [109]						SED	Moderate
Bell et al., 2017 [42]	RCT	928	No reported	12–13	17	PA, SED	Weak
Bogart et al., 2016 [49]	RCT	1368	49.1% B	12.2 ± 0.68	5	H	Weak
Bonhauser et al., 2005 [87]	Quasi - Ex	198	I = 45.9% G C = 57% G	I = 15.5 ± 0.8 C = 15.5 ± 0.9	43	PA, F, WB	Weak
Budde et al., 2010 [52]	RCT	59	I_1_ = 50% B I_2_ = 55% B C = 62% B	14.4 ± 0.5	12 min	H, AC	Weak
Bush et al., 2010 [126]	Quasi - Ex	191	I = 48% B C = 32% B	I = 13.9 ± 0.7 B I = 13.8 ± 0.9 G C = 12.5 ± 0.6 B C = 12.5 ± 0.6 G	16	PA	Weak
Laberge et al., 2012 [110]						AC	Weak
Carlin et al., 2018 [76]	RCT - cluster	197	100% G	11–13, 12.4 ± 0.6	12	PA, SED, H, F, WB	Moderate
Chen et al., 2020 [85]	Quasi - Ex	377	I_1_ = 53.6% G I_2_ = 55.4% G	No reported	12	PA	Weak
Contardo Ayala et al., 2018 [80]	Quasi - Ex	105	43.2% G	14.8 ± 1.7, 12–17	17	H, F	Weak
Sudholz et al., 2020 [100]						PA, SED, AC	Weak
Corder et al., 2016 [51]	RCT	788	I = 47.7% B C = 43.5% B	I = 13.2 ± 0.4 C = 13.1 ± 0.3	8	PA, WB	Moderate
Corder et al., 2020 [73]	RCT - cluster	2862	I = 46.6% G C = 48.9% G	I = 13.2 ± 0.4 C = 13.2 ± 0.4	12	PA, SED, H, WB	Weak
Corepal et al., 2019 [56]	RCT - cluster	224	46.9% B	12–14	22	PA, SED	Weak
Costigan et al., 2018 [46]	RCT	65	69% B	15.8 ± 0.6	8	PA	Weak
Cui et al., 2012 [53]	RCT	682	I = 50.9% B C = 52.7% B	I = 12.7 ± 0.5 B I = 12.6 ± 0.5 G C = 12.8 ± 0.5 B C = 12.6 ± 0.4 G	4	PA, SED	Weak
Gammon et al., 2019 [71]	RCT - cluster	222	51% B	No reported	12	PA, SED	Weak
Ghammam et al., 2017 [81]	Quasi - Ex	4003	I = 50.2% B C = 46.5% B	11–16	156	PA, SED	Weak
Haapala et al., 2017 [86]	Quasi - Ex	319	66.7% G	14.0 ± 0.6	87	PA, SED	Weak
Haerens et al., 2006 [35]	RCT - cluster	2840	36.6% G	13.1 ± 0.8, 11–15	43	PA, H	Moderate
Haerens et al., 2007 [98]						PA	Moderate
Harrington, 2018 [36]	RCT - cluster	1752	100% G	11–14	61	PA, SED	Strong
Gorely et al., 2019 [115]						FRI	Strong
Hollis et al., 2016 [60]	RCT - cluster	1150	48% B	12	104	H	Strong
Sutherland et al., 2016 [102]						PA, FRI	Strong
Sutherland et al., 2016 [127]						PA, FRI	Moderate
Sutherland et al., 2020 [34]	RCT - cluster	6476	No reported	No reported	52	FRI	Weak
Sutherland et al., 2021 [45]					104	FRI	Weak
James et al., 2020 [48]	RCT	909	51.2% B 48.8% G	13–14	52	H, F	Weak
Kariippanon et al., 2019 [43]	Cross-over trial	171	No reported	13.2 ± 1	2	PA, SED	Weak
Kennedy et al., 2018 [77]	RCT - cluster	607	50.1% G	14.1 ± 0.5	10	F, PA, FRI	Weak
Kennedy et al., 2019 [117]						FRI	Weak
Kennedy et al., 2021 [92]	Hybrid	750	47.6% G	14.4 ± 1	10	F, FRI	Weak
Knebel et al., 2020 [68]	RCT - cluster	597	I = 51% G C = 54.1% G	13.0 ± 1.0	39	SED	Weak
Knox et al., 2012 [84]	Quasi – Ex	192	No reported	I = 12.4 ± 0.5 C = 12.1 ± 1.1	18	PA, H	Weak
Kolle et al., 2020 [65]	RCT - cluster	2084	I_1_ = 50% G I_2_ = 49% G C = 49% G	14 ± 0.3	39	PA, SED, F	Strong
Solberg et al., 2021 [78]						AC	Weak
Lazorick et al., 2015 [90]	Quasi - Ex	362	I = 53% B C = 51% B	I = 13.3 ± 0.8 C = 13.1 ± 0.5	14	PA, H	Weak
Leme et al., 2016 [107]	RCT - cluster	253	100% G	15.61 ± 0.05	26	PA, SED, H	Weak
Leme et al., 2018 [37]						PA, SED, H	Weak
Lubans et al., 2010 [93]	RCT	108	I_1_ = 59% B I_2_ = 49% B C = 47% B	15.0 ± 0.7	8	H, F	Weak
Lubans et al., 2011 [104]	RCT	100	100% B	14.3 ± 0.6	26	PA, H	Moderate
Morgan et al., 2012 [114]						WB	Weak
Lubans et al., 2012 [50]						PA, H	Moderate
Lubans et al., 2012 [118]	RCT - cluster	357	100% G	13.2 ± 0.5	52	PA, SED, H, F	Strong
Dewar et al., 2013 [59]						PA, SED, H	Strong
Dewar et al., 2014 [103]						PA, SED, AC	Strong
Lubans et al., 2021 [99]	RCT - cluster	670	44.6% G	16.0 ± 0.43	52	PA, H, AC, F, WB	Moderate
Mavilidi et al., 2020 [66]	RCT - cluster	221	49.8% G	16.0 ± 0.5 16–18	52	AC	Weak
Valkenborghs et al., 2022 [113]	RCT - cluster	56	61% G	16.1 ± 0.4	26	H	Strong
Ludyga et al., 2018 [64]	RCT - cluster	36	I = 12% G C = 24.2% G	12–15	8	AC	Weak
Ludyga et al., 2019 [62]	RCT - cluster	94	100% B	13.9 ± 0.8, 12–15	20 min session	AC	Weak
Melnyk et al., 2013 [67]	RCT - cluster	779	51.5% G	14–16	26	PA, H, AC	Weak
Melnyk et al., 2015 [111]						H, WB	Weak
Murphy et al., 2022 [41]	Quasi - Ex	85	100% G	13 ± 0.7	10	PA, WB, F	Weak
Okely et al., 2017 [38]	RCT - cluster	1769	100% G	13.6 ± 0.0	78	PA, SED	Strong
Parrish et all, 2018 [57]	RCT - cluster	88	50% B	14.7 ± 0.7, 13–16	22	PA, SED, AC	Moderate
Peralta et al., 2009 [33]	RCT	33	100% B	12.5 ± 0.4	26	PA, H, F	Weak
Schofield et al., 2005 [40]	Quasi - Ex	90	100% G	15.8 ± 0.8, 15–18	12	PA, H	Weak
Sebire et al., 2018 [39]	RCT - cluster	427	100% G	12–13	22	PA	Moderate
Sebire et al., 2019 [116]						FRI	Moderate
Smith et al., 2014 [106]	RCT - cluster	361	100% B	12.7 ± 0.5	20	PA, SED, H	Weak
Lubans et al., 2016 [108]						PA, SED	Weak
Lubans et al., 2016 [69]						SED, H, F, WB	Weak
Subramanian et al., 2015 [47]	RCT	439	43.1% G	12–17	26	AC	Weak
Suchert et al., 2015 [61]	RCT - cluster	1162	48% G	13.7 ± 0.7, 12–17	12	PA, SED, F	Moderate
Sudholz et al., 2016 [79]	Quasi - Ex	43	49% G	13.7 ± 1.4, 12–16	7	PA, SED	Weak
Tarp et al., 2016 [72]	RCT - cluster	632	48.9% G	12.9 ± 0.6	20	PA, H, AC, F	Moderate
Torbeyns et al., 2017 [44]	RCT	56	51.8% B	14.3 ± 0.6	22	H, F	Weak
Tymms et al., 2016 [58]	RCT - cluster	1494	I = 51.0% G C = 53.3% G	I = 11.72 C = 11.79	6	PA	Weak
Van Woudenberg et al., 2018 [75]	RCT - cluster	190	53.68% G	12.17, 11–14	1	PA	Weak
Verloigne et al., 2018 [74]	RCT - cluster	156	54.5% G	15.5 ± 0.5	26	PA, SED, WB	Moderate
Yang et al., 2017 [83]	Quasi - Ex	820	I = 73.2% B C = 79.1% B	I = 10.9 ± 1.6 C = 11.0 ± 1.5	52	PA, H, F	Weak
Yli-Piipari et al., 2016 [82]	Quasi - Ex-	94	51.1% G	11–15	4	PA, F	Weak
Yu et al., 2021 [91]	Quasi - Ex	514	No reported	No reported	21	PA, FRI	Weak

Abbreviations: n = number; y = years. * (Study design) RCT = randomised controlled trial; Quasi - ex = quasi experimental; Hybrid = hybrid effectiveness-implementation trial design

^**†**^ (Sex) B = boys; G = girls; I = Intervention group; C = comparison/control group ^**‡**^ (Outcomes) PA = physical activity; SED = sedentary behaviour; WB = wellbeing; H = health; F = fitness, AC = academic outcomes; FRI = factors relating to the implementation

### Strategies Used within Secondary-School Interventions and their Effect on Movement Behaviours, Cognitive/Academic, Physical Health and/or Psychological Outcomes

Various unique intervention strategies were reported (*n* = 23) across 11 categories (i.e., active lessons, community involvement, educational resources, environmental, incentives/rewards, peer support, physical activity session/s, research support, school policy, teacher training, and technology strategies). A definition, detailed description, and examples of these categories can be found in Table 2 and Supplementary Table 2, respectively. Single component interventions using only one strategy (*n* = 19 studies, 28.6%) involved physical activity sessions (*n* = 10), peer-led support (*n* = 6), or an environmental modification strategy (*n* = 3). Most interventions (*n* = 45 studies, 71.4%) involved the use of a combination of two or more strategies (‘*multicomponent interventions*’). The strategies most frequently used were: physical activity sessions (*n* = 31); environmental modifications (*n* = 29); educational resources (*n* = 25 interventions); peer support (*n* = 20); teacher training (*n* = 18); supporting technology (*n* = 11); active lessons (*n* = 8); community involvement (*n* = 7); research support (*n* = 6); incentives/rewards (*n* = 4); and school policies (*n* = 3) (Table 3). Although some interventions targeted several elements of the school day (e.g., class, recess/lunch, homework), 29 studies (46%) included a class-time component [37, 43, 44, 46, 50–55, 57–59, 63, 64, 66–72, 74, 79, 80, 84, 87, 89, 90], 22 studies (34.9%) a recess/lunch component [34, 35, 37, 38, 46, 49, 56, 57, 63, 65, 68–70, 72, 76, 77, 83, 86, 88, 91–94], 15 (23.8%) studies a whole-school day approach [36, 39, 40, 50, 56, 57, 61, 63, 68, 69, 72, 73, 75, 81, 83, 95], and one intervention (1.6%) [96] included an active homework component. Table 4 summarizes studies that report a positive impact on movement behaviours, cognitive/academic performance, physical wellbeing, and psychological outcomes, sorted by their methodological quality. These interventions encompassed a range of strategies, including physical activity sessions, environmental enhancements, teacher training, peer support, and educational resources.

**Table 2:** Definitions of the intervention strategies reported

Name of the strategy	Definition
**Active lessons**	Teachers’ normal planned class lessons, where the delivery method rather than the content is changed (PA into curriculum subjects)
**Community involvement**:	Participation/support of community members/facilities outside schools
**Educational**	Theory based sessions only (health-related)
**Environmental modifications**:	A supportive school environment encourages physical activity throughout the school day (e.g. active indoor and outdoor environments, active equipment)
**Incentives/rewards**:	Incentives to promote or reward physical activities or certain goals
**Physical activity sessions**	Opportunities for PA (supervised or unsupervised) during schools’ hours (e.g. during recess and lunchbreak)
**Peer support**:	Adolescent leaders to encourage PA among their peers
**Research support**:	Direct support from the research team involved
**School policy**	Changes, adaptation of the school policies to encourage physical activity and reduce sitting time
**Teacher training**	Teacher development sessions, pedagogical strategies

**Abbreviations: PA = physical activity**

**Table 3:** Frequency of intervention strategies used across studies

Strategies used	References
PA session	[33, 34, 38, 41, 46, 47, 54, 60, 62, 64–67, 71, 72, 77, 82–84, 86, 90, 92, 94, 95, 99, 104, 106, 107, 112, 113, 118]
Environment	[34, 35, 40, 43, 44, 49, 55–57, 66, 68, 70, 74, 77, 79–81, 83, 85, 89, 91, 95, 99, 104, 106, 113, 118, 125]
Educational	[33, 38, 43, 44, 47, 54, 57, 66, 74, 76, 78, 79, 81, 86, 89, 90, 92, 94–96, 101, 112, 116, 117, 127]
Peer support	[36, 39, 42, 48, 49, 51, 53, 55, 56, 58, 60, 67, 70, 73, 75, 76, 81, 104, 106, 126]
Teacher training	[36, 43, 57, 58, 60, 63, 66–68, 74, 77, 80, 85, 90, 99, 107, 113, 117, 118]
Technology	[55, 56, 61, 72, 77, 92, 99, 106, 113, 118]
Community involvement	[41, 76, 86, 94, 101, 115, 116, 127]
Active lessons	[52, 63, 65, 80, 87, 89, 126]
Research support	[41, 43, 45, 52, 115, 117]
Reward/ Incentives	[33, 45, 51, 55]
School policy	[41, 115, 127]

**Table 4:** Studies that reported a positive significant effect on movement behaviours, academic outcomes, physical health and/or psychological outcomes and methodological quality of the studies

	Methodological quality	Movement behaviour			Academic outcomes	Physical Health						Psychological outcomes
Study	Overall Rating	PA	SED	EE		BMI	WC	BF	Cholesterol	BP	Fitness	
Dewar et al., 2013 [59]	Strong		✓					✓				
Harrington et al., 2018 [36]	Strong	✓	✓									
Hollis et al., 2016 [60]	Strong					✓						
Lubans et al., 2012 [118]	Strong		✓									
Sutherland et al., 2016 [102]	Strong	✓										
Aceves-Martins et al., 2017 [55]	Moderate	✓	✓									
Andrade et al., 2014 [70]	Moderate										✓	
Barbosa Filho et al., 2016 [63]	Moderate	✓										
Barbosa Filho et al., 2019 [97]	Moderate	✓										
Carlin et al., 2018 [76]	Moderate	✓	✓									✓
Corder et al., 2020 [73]	Moderate	✓										
Haerens et al., 2007 [98]	Moderate	✓										
Haerens et al., 2006 [35]	Moderate							✓				
Lubans et al., 2011 [104]	Moderate					✓		✓				
Lubans et al., 2012 [50]	Moderate					✓		✓				
Lubans et al., 2021 [99]	Moderate	✓									✓	
Parrish et all, 2018 [57]	Moderate	✓			✓							
Sebire et al., 2019 [116]	Moderate	✓										
Suchert et al., 2015 [61]	Moderate	✓										
Sutherland et al., 2016 [127]	Moderate	✓										
Tarp et al., 2016 [72]	Moderate					✓						
Ahmed et al., 2022 [95]	Weak	✓	✓									
Altunkurek et al., 2019 [54]	Weak											✓
Amoah et al., 2021 [94]	Weak	✓				✓				✓		
Ardic et al., 2016 [89]	Weak	✓										
Bogart et al., 2016 [49]	Weak					✓						
Bonhauser et al., 2005 [87]	Weak										✓	✓
Budde et al., 2010 [52]	Weak				✓							✓
Chen et al., 2020 [85]	Weak	✓										
Contardo Ayala et al., 2018 [80]	Weak			✓			✓					
Corder et al., 2016 [51]	Weak											✓
James et al., 2020 [48]	Weak									✓		
Kariippanon et al., 2019 [43]	Weak	✓	✓									
Kennedy et al., 2018 [77]	Weak										✓	
Kennedy et al., 2021 [92]	Weak	✓										
Knox et al., 2012 [84]	Weak						✓		✓	✓		
Laberge et al., 2012 [110]	Weak				✓							
Lazorick et al., 2015 [90]	Weak					✓						
Leme et al., 2016 [107]	Weak		✓									
Lubans et al., 2010 [93]	Weak					✓		✓				
Lubans et al., 2016 [108]	Weak		✓									
Lubans et al., 2016 [69]	Weak		✓								✓	✓
Ludyga et al., 2018 [64]	Weak				✓							
Ludyga et al., 2019 [62]	Weak				✓							
Mavilidi et al., 2020 [66]	Weak				✓							
Melnyk et al., 2013 [67]	Weak	✓			✓	✓						
Melnyk et al., 2015 [111]	Weak					✓						✓
Morgan et al., 2012 [114]	Weak											✓
Murphy et al., 2022 [41]	Weak											✓
Schofield et al., 2005 [40]	Weak	✓										
Smith et al., 2014 [106]	Weak		✓									
Solberg et al., 2021 [78]	Weak				✓							
Subramanian et al., 2015 [47]	Weak				✓							
Sudholz et al., 2016 [79]	Weak	✓	✓									
Sudholz et al., 2020 [100]	Weak	✓	✓		✓							
Torbeyns et al., 2017 [44]	Weak			✓								
Yang et al., 2017 [83]	Weak									✓	✓	
Yu et al., 2021 [91]	Weak	✓										

Legend: ✓ indicates a study that reported a positive significant effect on movement behaviours, academic outcomes, physical health and/or psychological outcomes

### Intervention Effects on Movement Behaviours

Sixty-three articles (74%) described intervention effects on *movement behaviours* (self-reported and/or device measured) (Table 1, outcomes). Of these, 40 articles (47%) reported on device-measured physical activity (i.e., light- [LPA], moderate- [MPA], vigorous- [VPA], moderate- to vigorous-intensity [MVPA] physical activity). Sedentary time was reported in 31 articles (36.4%); of these, 20 (23.5%) used device-measured sedentary/sitting time. A descriptive summary of the number of intervention strategies and their impact on different outcomes is presented in Supplementary Table 3.

**Fig. 2:**
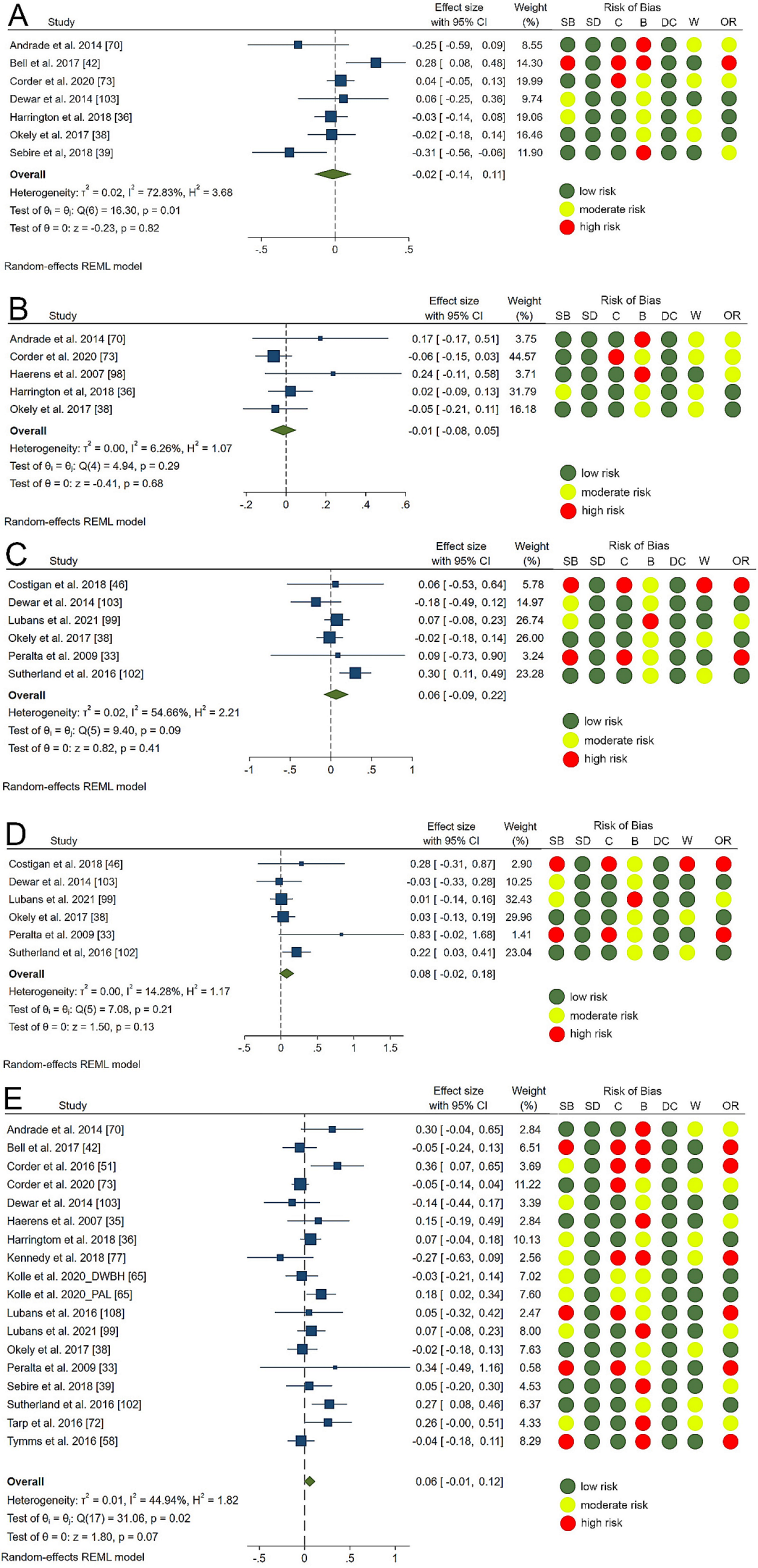
Forest plots for the effect of school-based initiatives on device measured sedentary time (**A**), light intensity physical activity (**B**), moderate physical activity (**C**), vigorous physical activity (**D**), moderate-to-vigorous physical activity (**E**). Legend: Risk of bias: (SB) selection bias; (SD) study design; (C) confounder; (B) blinding; (DC) data collection; (W) withdrawal and (OR) overall risk

Sufficient data for meta-analysis were available for sedentary time, LPA, MPA, VPA, MVPA assessed during the whole day (Summary statistics used for calculation of standardized mean difference across the studies are presented in Supplementary Table 7). All meta-analyses showed non-significant effects: Sedentary time, SMD = -0.02 (95% CI: -0.14, 0.11), Fig. 2.A; LPA, SMD = -0.01 (95% CI: -0.08, 0.05), Fig. 2.B; MPA, SMD = 0.06 (95% CI: -0.09,0.22), Fig. 2.C; VPA, SMD = 0.08 (95% CI: -0.02, 0.18), Fig. 2.D; MVPA, SMD = 0.05 (95% CI: -0.01, 0.12), Fig. 2.E).

### Intervention Effects on Cognitive and Academic Outcomes

*Cognitive and academic outcomes* were measured in 12 articles (14.1%) which did not fulfill the inclusion criteria for meta-analyses. The most frequently explored outcomes were executive functions. Evidence of a positive impact was reported on working memory [52, 57, 64], inhibitory control [62], cognitive flexibility [47], verbal fluency [47], attention and concentration [47, 110], and on-task behaviour [66]. No effects were found on inhibitory control [72, 99], and working memory [99]. A positive intervention effect was found on health course grades [67], numeracy and reading performance [78] and academically-relevant social skills ratings (cooperation, assertion, academic competence) [67]. No effect was found on mathematical skills [72].

### Intervention Effects on Physical Health Outcomes

**Fig. 3:**
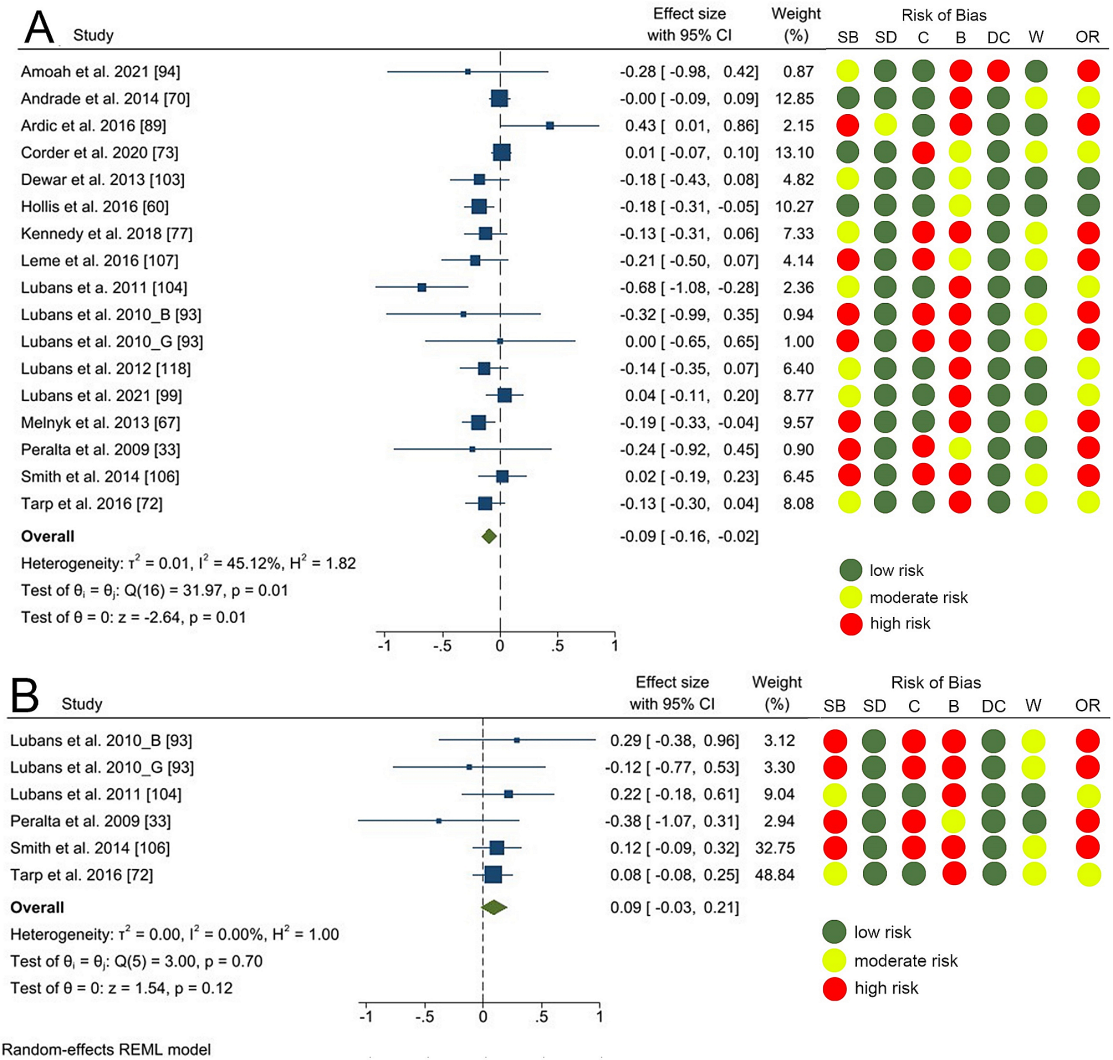
Forest plots for the effect of school-based initiatives on body mass index (**A**) and waist circumference (**B**). Legend: Risk of bias: (SB) selection bias; (SD) study design; (C) confounder; (B) blinding; (DC) data collection; (W) withdrawal and (OR) overall risk

*Physical health related outcomes* were assessed in 29 articles (34.1%). Meta analysis of 16 articles (18.8%) showed a statistically significant small effect on BMI (SMD= -0.09 [95% CI: -0.16, -0.02]) with a moderate level of heterogeneity and 56% of the articles were assessed with high-risk of bias; Fig. 3.A). For waist circumference, 5 articles (5.9%) provided necessary data for meta-analysis. A non-significant intervention effect was found (SMD = 0.09 [95% CI: -0.03, 0.21]; Fig. 3, B).

Evidence of a positive effect was reported for blood pressure [48, 83, 84, 94], high-density lipoprotein (HDL) to total cholesterol ratio [84], and glucose [84]. No effects on body fat [33, 106], blood pressure [76] or musculoskeletal health [80] were found. Fitness-related outcomes were assessed in 19 articles (22.4%). Positive effects on cardiorespiratory fitness (e.g. increase distance run) [44, 48, 65, 72, 87, 92, 99], and muscular fitness (e.g. muscular endurance strength) were found [69, 70, 83, 87, 99]. No effects on cardiorespiratory fitness [33, 41, 61, 76, 82] and muscular fitness [41, 50, 65, 82, 112] were reported. Hippocampal metabolism was assessed [113], showing a significant intervention effect on N-acetylaspartate and glutamate + glutamine in the left hippocampus.

### Intervention Effects on Psychological Outcomes

*Psychological outcomes* were assessed in 12 articles (14.1%), which did not fulfill the inclusion criteria for meta-analyses. Positive effects were found in: anxiety and self-esteem [87], social support [51, 76], wellbeing [41, 51, 54, 69], self-perception [114], and lower depressive symptoms in participants with elevated depressive symptoms at baseline [111]. No effects on wellbeing [73, 99] or on stress and internalization/externalization problems [99] were reported, and one study indicated that the relationship with classmates deteriorated in the intervention group [74]. Also, there were no effects on social cognitive variables for physical activity (i.e., self-efficacy, perceived environment, social support, behavioural strategies, outcome expectations and outcome expectancies related to physical activity) [103].

### Methodological Quality

Of the included articles, 65% received an overall weak quality rating, 25% were rated as moderate, and 11% were considered strong quality (Supplementary Table 4). A breakdown of the methodological quality per article is shown in Table 5. Study design, data collection methods, confounders, withdrawals and dropout were assessed as strong in > 50% of the articles. Selection bias was considered weak in 45% of the articles. For articles that had a positive effect on an outcome reported in this review (*n* = 58) [68%], 16 were classified as strong, 17 as moderate, and 25 as weak (Table 4).

**Table 5:** Methodological quality assessment for each article

Study	Selection bias	Study design	Confounder	Blinding	Data collection	Withdrawal	Overall Rating
Aceves-Martins et al., 2017 [55]	2	1	1	3	1	2	2
Ahmed et al., 2022 [95]	3	1	1	3	1	1	3
Altunkurek et al., 2019 [54]	3	1	1	3	3	3	3
Amoah et al., 2021 [94]	2	1	1	3	3	1	3
Andrade et al., 2014 [70]	1	1	1	3	1	2	2
Andrade et al., 2015 [96]	1	1	1	3	1	2	2
Ardic et al., 2016 [89]	3	2	1	3	1	1	3
Bandeira et al., 2020 [109]	1	1	1	3	1	1	2
Barbosa Filho et al., 2016 [63]	1	1	1	3	1	1	2
Barbosa Filho et al., 2017 [125]	1	1	1	3	1	1	2
Barbosa Filho et al., 2019 [97]	1	1	1	3	1	1	2
Bell et al., 2017 [42]	3	1	3	3	1	1	3
Bogart et al., 2016 [49]	2	1	1	3	1	3	3
Bonhauser et al., 2005 [87]	3	3	1	3	3	1	3
Budde et al., 2010 [52]	3	1	3	3	3	1	3
Bush et al., 2010 [126]	2	3	1	3	1	2	3
Carlin et al., 2018 [76]	2	1	1	3	1	1	2
Chen et al., 2020 [85]	3	3	3	3	2	2	3
Contardo Ayala et al., 2018 [80]	3	3	1	3	1	1	3
Corder et al., 2016 [51]	2	1	3	3	1	1	3
Corder et al., 2020 [73]	1	1	3	2	1	2	2
Corepal et al., 2019 [56]	2	1	3	3	1	1	3
Costigan et al., 2018 [46]	3	1	3	2	1	3	3
Cui et al., 2012 [53]	3	1	1	3	2	1	3
Dewar et al., 2013 [59]	2	1	1	2	1	1	1
Dewar et al., 2014 [103]	2	1	1	2	1	1	1
Gammon et al., 2019 [71]	3	1	3	3	1	1	3
Ghammam et al., 2017 [81]	1	3	1	3	3	1	3
Gorely et al., 2019 [115]	2	1	3	2	1	3	3
Haapala et al., 2017 [86]	3	3	1	3	1	3	3
Haerens et al., 2006 [35]	1	1	1	3	1	1	2
Haerens et al., 2007 [98]	1	1	1	3	1	1	2
Harrington, 2018 [36]	2	1	1	2	1	2	1
Hollis et al., 2016 [60]	1	1	1	2	1	1	1
James et al., 2020 [48]	2	1	3	3	1	1	3
Kariippanon et al., 2019 [43]	2	3	1	3	1	1	3
Kennedy et al., 2018 [77]	2	1	3	3	1	2	3
Kennedy et al., 2019 [117]	2	1	3	3	1	2	3
Kennedy et al., 2021 [92]	2	3	3	3	1	3	3
Knebel et al., 2020 [68]	3	1	1	3	3	2	3
Knox et al., 2012 [84]	3	3	1	3	1	3	3
Kolle et al., 2020 [65]	2	1	2	2	1	1	1
Laberge et al., 2012 [110]	2	3	3	3	1	2	3
Lazorick et al., 2015 [90]	2	3	1	3	1	2	3
Leme et al., 2016 [107]	3	1	3	2	1	2	3
Leme et al., 2018 [37]	3	1	3	2	1	2	3
Lubans et al., 2010 [93]	3	1	3	3	1	2	3
Lubans et al., 2011 [104]	2	1	1	3	1	1	2
Lubans et al., 2012 [118]	2	1	1	2	1	1	1
Lubans et al., 2012 [50]	2	1	1	3	1	1	2
Lubans et al., 2016 [108]	3	1	3	2	1	1	3
Lubans et al., 2016 [69]	3	1	3	2	1	1	3
Lubans et al., 2021 [99]	2	1	1	3	1	1	2
Ludyga et al., 2018 [64]	3	1	1	3	1	1	3
Ludyga et al., 2019 [62]	3	1	3	3	1	1	3
Mavilidi et al., 2020 [66]	2	1	3	3	1	1	3
Melnyk et al., 2013 [67]	3	1	1	3	1	2	3
Melnyk et al., 2015 [111]	3	1	1	3	1	2	3
Morgan et al., 2012 [114]	2	1	3	3	1	1	3
Murphy et al., 2022 [41]	3	3	3	3	3	3	3
Okely et al., 2017 [38]	1	1	1	2	1	2	1
Parrish et all, 2018 [57]	3	1	1	2	1	1	2
Peralta et al., 2009 [33]	3	1	3	2	1	1	3
Schofield et al., 2005 [40]	2	3	1	3	1	2	3
Sebire et al., 2018 [39]	1	1	1	3	1	1	2
Sebire et al., 2019 [116]	1	1	1	3	1	1	2
Smith et al., 2014 [106]	3	1	3	3	1	2	3
Solberg et al., 2021 [78]	2	1	3	3	1	1	3
Subramanian et al., 2015 [47]	3	1	1	3	3	2	3
Suchert et al., 2015 [61]	1	1	1	3	1	1	2
Sudholz et al., 2016 [79]	3	3	3	3	1	3	3
Sudholz et al., 2020 [100]	3	3	1	3	1	1	3
Sutherland et al., 2020 [34]	3	1	1	3	1	1	3
Sutherland et al., 2021 [45]	3	1	3	3	1	1	3
Sutherland et al., 2016 [102]	1	1	1	2	1	2	1
Sutherland et al., 2016 [127]	1	1	3	2	1	2	2
Tarp et al., 2016 [72]	2	1	1	3	1	2	2
Torbeyns et al., 2017 [44]	3	1	3	3	1	1	3
Tymms et al., 2016 [58]	3	1	1	3	1	1	3
Valkenborghs et al., 2022 [113]	2	1	1	2	1	1	1
Van Woudenberg et al., 2018 [75]	3	1	1	3	1	1	3
Verloigne et al., 2018 [74]	2	1	1	3	1	1	2
Yang et al., 2017 [83]	3	3	3	3	3	1	3
Yli-Piipari et al., 2016 [82]	3	1	3	3	1	3	3
Yu et al., 2021 [91]	3	3	3	3	2	3	3

Legend: 1 = strong, 2 = moderate, 3 = weak methodological quality

### Factors Relevant to the Implementation

At least one *factor relevant to the implementation* of the programs (implementation, mechanism of impact and/or context) was reported in 43 articles (50.5%). Specific components reported included *fidelity* (*n* = 26, 30.5%) [33, 34, 38, 45, 53, 57, 58, 60, 63, 66, 70, 73, 77, 82, 92, 99, 101, 102, 105, 107, 111, 115–117]. Fidelity was assessed based on adherence to the intervention protocol and consistency in delivery, of which 14 articles (16.4%) indicated that interventions were delivered as planned (> 60% of consistency with the original plan); *participant responses* (*n* = 25, 29.4%)) [33, 38, 46, 51, 53, 55–57, 67, 70, 73, 74, 77, 79, 88, 92, 101, 106, 107, 111, 115–117], where 20 articles (23.5%), indicated the majority of the participants enjoyed or participated in the intervention; *dose* (*n* = 18, 21.2%) [34, 45, 48, 57, 60, 63, 66, 70, 73, 77, 85, 87, 99, 101, 102, 105, 115, 117], of which 10 indicated that the intervention was delivered and received as planned (> 50% of the frequency and duration of the intervention was delivered); *reach* (*n* = 18, 21.2%) [33, 34, 45, 55, 60, 63, 64, 70, 88, 92, 93, 106, 107, 115–117], where 13 indicated that the intervention targeted the expected audience; *context* (*n* = 15, 17.6%) [33, 38, 42, 47, 48, 51, 74, 87, 99, 107, 115, 118], of which 4 indicated no adverse effects of the intervention, and 5 articles showed that the intervention needed some modifications (e.g. different devices, time for planning, resources); and *adaptation* was required in 6 studies (i.e. content and frequency adaptations) [34, 45, 63, 74, 77, 117].

### Publication Bias

Funnel plots for MVPA, BMI and WC are presented in Supplementary material 6, and show little sign of asymmetry. In addition, the Egger’s tests for all three outcomes were not statistically significant, indicating absence of small-sample effects. There was little evidence of publication bias with pooled effect size estimates using the trim-and-fill method similar to the main findings for MVPA (SMD = 0.06 vs. 0.05), with overall interpretations of effects unchanged for BMI (SMD=-0.08 vs. -0.08) and WC (SMD = 1.46 vs.1.46).

## Discussion

Our systematic review provided a narrative synthesis of the intervention strategies used within the secondary school settings to improve students’ movement behaviours throughout school-based initiatives and the factors related to intervention implementation, and a meta-analysis (where possible) of the effectiveness of school-based initiatives to increase physical activity and reduce sedentary time on adolescents’ movement behaviours, energy expenditure, cognitive/academic, behavioural and physical and psychological health outcomes. Intervention strategies that reported favourable effects on movement behaviours, cognitive/academic, physical and psychological outcomes tended to include physical activity sessions, environmental modifications, teacher training, peer support and/or educational resources. Despite some promising findings for BMI, the meta-analysis showed no significant effects of interventions on the total accumulated daily movement behaviours, assessed using accelerometers, and waist circumference.

Interventions incorporated a wide variety of strategies, with the majority being multicomponent. Previous reviews have concluded multicomponent interventions elicit greater effects on youth health and wellbeing compared to single component interventions [119], particularly those that use a whole-school approach [9]. However, it can be difficult to establish which strategies embedded within multicomponent interventions are most effective and may be context specific. Homework and recess time were seldom targeted in interventions. This is consistent with a recent review and meta-analysis of school-based recess interventions, which reported that just one of 43 interventions targeted secondary school students [120]. Physical activity sessions, educational resources, environmental modifications, peer-support, and teacher training were the most commonly used strategies, consistent with a previous review of school-based interventions to increase adolescent physical activity [16]. Although active lessons are commonly used and can have positive effects on lesson-time physical activity in primary/elementary school interventions [121], only eight of the 63 interventions in this review incorporated active lessons. There is clearly a need for evidence of the effectiveness of interventions involving active lessons, recess and lunch breaks and active homework.

Our meta-analyses revealed small positive effects of overall interventions on BMI; however, most articles were assessed with a high risk of bias. Results related to BMI are consistent with findings from a previous review indicating that school-based physical activity interventions result in a very small decrease in BMI z-score in children and adolescents [23]. We did not find significant effects for accelerometer-determined physical activity of any intensity. This is consistent with previous meta-analyses, which found that school-based physical activity interventions had small or no benefits on whole-day moderate-intensity physical activity levels of adolescents [15–17]. This review found few studies reported the impact on sedentary time and LPA, especially during school hours. It found a statistically significant effect for sitting and stepping time (data not shown, as two out the four studies reporting these data did not meet the inclusion criteria for analysis [i.e. randomised controlled trials]). The effect of school-based initiatives on movement behaviours may have been under-estimated as these initiatives could be more effective at reducing sedentary time and increasing LPA rather than higher intensity activity during school hours. Future research should consider assessing multiple movement behaviour intensities (i.e., sedentary time, LPA) with the appropriate devices [122].

The quality assessment of the studies showed different distributions of effectiveness between weak, moderate and strong quality. Further analyses showed no apparent differences in quality between studies that reported significant effects and those that did not for each outcome; however, only few studies were classified with low risk of bias. Only 43 articles (50.5%) reported factors relevant to the implementation of the programs. Therefore, with the lack of and inconsistencies in reporting implementation-relevant information, it is difficult to determine whether the interventions were sufficiently or appropriately implemented as intended. Insufficient equipment or supportive environments (e.g., standing desks), high dropout rates due to academic commitments, cancelled sessions due to unplanned activities, lack of time to plan and deliver, and programs not viewed as a priority were reported as factors that could influence intervention effectiveness. Evaluating factors related to implementation before and during the intervention, that allows researchers and schools to adapt and improve the implementation strategies to the school’s and students’ needs, could achieve better results [123].

Limitations of included studies were the high level of heterogeneity between outcome measures, and that only a small number of studies reported movement behaviours during school hours. It could be possible that interventions were effective during school hours, but effects were compensated for outside the school [124]. Future school-based interventions should consider (a) incorporating whole of school approaches (b) including additional outcomes such as academic performance and reporting movement behaviour outcomes for school hours and the whole day and multiple intensities, (c) reporting factors relevant to the implementation to assist in interpreting effectiveness.

Schools should be encouraged to develop and implement policies that support whole-of-school physical activity strategies. These policies could include providing teaching relief so teachers can receive professional development (e.g., to deliver active lessons in the classroom), and allocating space in the curriculum for additional physical activity sessions and delivery of educational resources.

## Conclusions

While some intervention approaches for increasing adolescents’ physical activity and reducing sedentary time in secondary schools demonstrated promise (e.g. physical activity sessions, environmental modifications, teacher training, peer support, educational resources and/or active lesson strategies), small or non-significant effects were found in the meta-analyses. Future movement behaviour interventions in secondary schools should utilize a whole-school approach to beneficially change adolescents’ activity levels. Consistent reporting of implementation will increase understanding of how interventions effect outcomes.

### Electronic Supplementary Material

Below is the link to the electronic supplementary material.

Supplementary Material 1

## Data Availability

The datasets used and/or analyzed in the current study will be supplied by the corresponding author upon reasonable request.

## Abbreviations

SMD: Standardized mean difference
CI: Confidence interval
MVPA: Moderate- to vigorous-intensity physical activity
PE: Physical education
PICOS: Participants, Interventions, Comparisons, Outcomes, and Study design framework
RCTs: Randomized controlled trials
PRISMA: Preferred Reporting Items for Systematic Reviews and Meta-Analyses
LPA: Light physical activity
MPA: Moderate physical activity
VPA: Vigorous physical activity
BMI: Body mass index
HDL: High-density lipoprotein
N: Number
SED: Sedentary behaviour
EE: Energy expenditure
WC: Waist circumference
CRF: Cardiorespiratory fitness
SB: Selection bias
SD: Study design
C: Confounder
B: Blinding
DC: Data collection
W: Withdrawal
OR: Overall risk
