# Supplementary material for: Effectiveness of Intervention Strategies to Increase Adolescents’ Physical Activity and Reduce Sedentary Time in Secondary School Settings, Including Factors Related to Implementation: A Systematic Review and Meta-Analysis
Source: Sports Med Open. 2024 Mar 13;10:25. doi: 10.1186/s40798-024-00688-7 (PMC10933250; doi:10.1186/s40798-024-00688-7)
Supplement: Supplementary file 1 — Supplementary Material 1 [file 40798_2024_688_MOESM1_ESM.docx]

## Supplementary Table 1:

| **Search strategy used** | **EBSCOHost: MEDLINE complete, CINAHL, SPORTDiscus, APA PsycINFO, and ERIC** |
| --- | --- |
| **Population** | adolescence* OR teen* OR youth OR pupils OR child* OR "secondary student" OR "high school student" OR student* OR “young people" |
| **Setting** | school OR “secondary college” OR “school-based” OR “secondary education” OR class* OR lesson* OR “classroom lesson*” OR "classroom-based" OR "school-based" OR "classroom exercise" OR “class time” OR “lesson time” OR “homework” |
| **Intervention** | break* OR “active break” OR physical* OR activ* OR “physical activity” OR “stand* desk*” OR “height adjustable desk*” OR “dynamic seating” OR “class* environ*” OR “sedentary behavi*” OR sedentar* OR “sed* time” OR “sed* time” OR “sitting time” OR sit* |
| **Design** | “Randomi?ed controlled trial” OR “Controlled trial” OR “Pre and post” OR “Quasi-experimental” |
| **Search strategy used** | **EMBASE** |
| **Population** | (adolescen* OR teen* OR youth OR pupils OR child* OR 'secondary student' OR 'high school student' OR student* OR 'young people') |
| **Setting** | (school OR 'secondary college' OR 'school-based' OR 'secondary education' OR class* OR lesson* OR 'classroom lesson*' OR 'classroom-based' OR 'school-based' OR 'classroom exercise' OR 'class time' OR 'lesson time' OR 'homework') |
| **Intervention** | (break* OR ‘active break’ OR physical* OR activ* OR ‘physical activity’ OR ‘stand* desk*’ OR ‘height adjustable desk*’ OR ‘dynamic seating’ OR ‘class* environ*’ OR ‘sedentary behavi*’ OR sedentar* OR ‘sed* time’ OR ‘sed* time’ OR ‘sitting time’ OR sit*) |
| **Design** | (‘Randomi?ed controlled trial’ OR ‘Controlled trial’ OR ‘Pre and post’ OR Quasi-experimental) |

## Supplementary Table 2: Description and examples of the intervention strategies

| **Strategy** | **Description** |
| --- | --- |
| **Active lessons:** are teacher's normal planned class lessons, where the delivery method rather than the content is changed (PA into curriculum subjects) | |
| Know, 2012 | - Walking lesson (brisk walking) with learning stations, short talks (subject related), on stop stations (each 400 or 800 meters). 1 min/stations: worksheets, question/answers |
| Kolle, 2020 | - 30 min/week lesson of physically active learning   where play-based activities were integrated into other  curriculum subjects (i.e. math, English, Norwegian) |
| Tarp, 2016 | - Daily physical activity in academic subjects - PA homework (daily) |
| Gammon, 2019 | - Physically active lesson training was delivered to teachers over two after-school sessions: how to integrate movement into lessons; strategies included students collecting data from the environment for class activities and completing activities posted on classroom walls, instead of sitting at desks. |
| **Community involvement:** participation/support of community members/facilities outside schools | |
| Andrade, 2014,  Andrade 2015 | - Parent’s involvement: Parent workshops in line with educational package content |
| Chen, 2020 | - Family component: Monthly packets containing behavioural tools were provided to assist parents and children in modifying their behaviou*rs* |
| Dewar, 2013,  Dewar, 2014,  Lubans, 2012 | - Parent newsletters |
| Haerens, 2006,  Haerens, 2007 | - interactive meeting on PA-health - Home correspondence, - Free CD with the adult computer-tailored - intervention for PA to complete at home |
| Hollis, 2016,  Sutherland, 2016a  Sutherland, 2016b  Sutherland, 2020  Sutherland, 2021 | - Physical activity newsletter sent to parents |
| Lubans 2016b | - Parental strategies to reduce screen time: 4 newsletters   consequences of excessive screen use, strategies to reduce screen time at home, tips to avoid conflict |
| Okely, 2017 | - home/school/community links: How to promote school activity during sport sessions (e.g., use of local facilities, teacher identify and promote activities available in the local community). Local communities were asked to offer classes and incentives for the girls to participate. |
| **Educational:** Theory based sessions only (health-related) | |
| Ahmed et al, 2022 | - Health education session, (10 min/1/week) that were delivered in classroom by the researcher. - The content focussed on PA, SB, and healthy eating behaviours - educational materials (infographics) to the students to take home for their parents and other family members in promoting an active lifestyle. The “infographic” included information on benefits of PA, recommended PA levels, healthy eating, and screen-based behaviours including their health consequences. |
| Altunkurek, 2019 | - Group education: once/week for 45-60min (12 course hours). The topics included: healthy nutrition, stress management, anger management, internet and technology addiction, and study skills. teachers. Students were given homework and their practices discussed the following week. |
| Amoah et al, 2021 | - The interactive health education sections, the researchers did education on CVDs, its risk factors, causes, development and prevention among others in the intervention schools (three times a week, approx. 1 hour) |
| Andrade, 2014,  Andrade 2015 | - Education delivered within regular school curricula using books with a focus on physical activity and sedentary behaviour |
| Ardic, 2016 | - To build healthy lifestyle and cognitive-behavioural: creating a healthy lifestyle, strategies to build self-esteem, stress management goal setting, effective communication, nutrition, and physical activity. |
| Barbosa Filho, 2016,  Barbosa Filho, 2017,  Barbosa Filho, 2019,  Bandeira, 2020 | - Health education: Exposition of materials produced in the classroom and PE classes (eg, posters). |
| Bell, 2017 | - For Peer-lead: Knowledge, skills, confidence to promote PA and healthy eating to their school year group. Role model for healthy behaviours. Support ‘follow-up sessions: 4 follow-up sessions to support and encourage peer supporters in their role and provide further information about the benefits of healthy eating and physical activity |
| Chen, 2020 | - Education provides an integrated concepts-based physical education lessons that help promote knowledge through movement experiences |
| Cui, 2012 | - Peer education: lessons integrated into existing health education courses and class meeting over four consecutive weeks |
| Dewar, 2013,  Dewar, 2014,  Lubans, 2012 | - Accredited dieticians delivered the nutrition workshops, and members of the research team delivered the interactive educational seminars and distributed text messages to reinforce and encourage targeted health behaviours. |
| Knebel, 2020 | - Informative Thematic flyers: (a) PA, (b) sedentary behaviour, (c) healthy eating, and (d) additional messages about PA and sedentary behaviour. Banners to be posted and displayed in schools: (a) PA, (b) SB, (c) healthy eating, (d) academic performance and its relationship with PA |
| Lazorick, 2015 | - Education delivered within regular school curricula aiming at improving knowledge about health-related underlying factors as well as behaviours, such as diet and PA. |
| Leme, 2018,  Leme, 2016 | - Education components including: Weekly nutrition and PA key messages (10 x 20 minutes), Nutrition and PA handbook, Interactive seminars, Nutrition workshops, Parents newsletter, WhatsApp text messages, Diet/PA diaries |
| Lubans, 2011,  Lubans, 2012b,  Morgan, 2012 | - PA/nutrition handbooks with home-challenges and interactive seminars: lifestyle behaviour- 3 x 30min |
| Lubans, 2016b,  Lubans, 2016a,  Smith, 2014 | - Seminars (3 x 20min) by research team with information about intervention components, behavioural messages, PA/SB/resistance guidelines and student leadership component. |
| Melnyk, 2013,  Melnyk, 2015 | - Teens received a COPE (Creating Opportunities for Personal Empowerment Healthy Lifestyles TEEN) Program manual with homework activities for each of the 15 sessions that reinforced the content and skills in the program (cognitive–behavioral skills building, Physical activity and nutrition information) |
| Parrish, 2018 | - Structured learning modules (6, 15 min) supporting behavioural change based on attention, retention, production, motivation by Personal Development Health and PE teachers. |
| Peralta, 2009 | - Theoretical components: promoting physical activity through increasing physical self-esteem and self-efficacy, - Behaviour modification techniques (e.g. group goals converting time spent in physical activity to kilometers to reach a specified destination. - 1 curriculum session (60min)/week |
| Schofield, 2005 | - Received a personal logbook: Information how to be active daily tips to overcome barries injuries prevention strategies - Education (Healthy eating, benefits of PA, barries for PA and time management, motivation and positive thinking. |
| Sebire, 2018,  Sebire, 2019 | - Attended to 3 day education program by study team. Training covered: gender inequalities in PA, PLAN-A concept, sessions, activities, resources, practice delivery. Peer-support training   (Received a manual,   - Girls training, Importance of PA, Identify by the peers as influence how to provide informal support to increase PA, inspiration and empowerment to be active. interpersonal skills to be a peer-supporter - Diffuse messages and norms about leading physically active lifestyles to their friends to encourage and support them to maintain or increase their physical activity |
| Suchert, 2015 | - Introducing competitions, giving and creating ideas how to integrate PA in everyday life, reflecting strategies to be more physically active. - The headmaster and entire teaching staff of participating schools as well as parents received elaborated information material. |
| Tymms, 2016 | - 6/week session during year 7 geography lessons, in classroom/computer room. - Used GIS, supported teaching materials allowed children to collect and interpret data about their own day-to-day PA . - To understand of how environment may influence their health and well-being. |
| Yang, 2017 | - Handout and Educational videos (Nutrition & exercise), 5-10min/day, at recess & lunch break. - exercise education = PA easily performed in short time as break at school/home (aerobic exercise, stretching and correction posture (2 min exercise in classroom) E.g. Step by step; Let’s dance; Good walking posture. |
| **Environmental modifications:** A supportive school environment encourages physical activity throughout the school day (e.g. Active indoor and outdoor environments, Active equipment) | |
| Ahmed et al, 2022 | - An activity bin (a large container with playing materials including soccer balls, cricket bat & balls, skipping ropes, badminton) was introduced and used during lunchtime activity sessions. |
| Andrade, 2014, Andrade, 2015 | - Modifications including line markings to provide a walking trail and posters |
| Barbosa Filho, 2016,  Barbosa Filho, 2017,  Barbosa Filho, 2019,  Bandeira, 2020 | - PA equipment, and banners explained the game rules. - Pamphlets distribution to students and parents (messages related to PA and SB). |
| Bogart, 2016 | - Posters promoting PA |
| Chen, 2020 | - Monthly packets containing behavioral tools were provided to assist parents and children in modifying their behaviors. |
| Contardo Ayala, 2018,  Sudholz, 2020 | - Height adjustable desks (replaced all traditional furniture) - classroom posters (how to use the desk, health benefits of breaking up sitting and tips to reduce sitting time during classroom time - desk stickers with a message to break-up classroom sitting every 15 minutes with 2 minutes of standing |
| Ghammam, 2017 | - Distribution of sports equipment |
| Haerens, 2006  Haerens, 20017 | - Sports materials: ropes, frisbees, balls, and beach ball sets. Available at: noon (6/10 schools), after school hours (3/10 schools), breaks (1/10) |
| Hollis, 2016,  Sutherland, 2016a  Sutherland, 2016b  Sutherland, 2020  Sutherland, 2021 | - Balls, hoops, and ropes. - Pedometers |
| Kariippanon, 2019 | - Flexible learning spaces: combination of standard- and double-sized classrooms (M = 83 m2), incorporated: grouped tables, standing workstations, ottomans, couches, and write-able tables and walls. mart-boards and whiteboard walls available around the room |
| Knebel, 2020 | - A newly painted volleyball court (revitalized old courts) - Sports equipment kit (rackets, jumping rope, balls to play basketball, soccer, and volleyball) was provided to each school for use in intervals, lunchtime, and after school. |
| Lubans, 2011,  Lubans, 2012b,  Morgan, 2012 | - Pedometers for self-monitoring |
| Lubans, 2021,  Mavilidi, 2020 | - Equipment package ($AUS 2500) including:   - 1 × heart rate monitor per student,   - 1 × Bluetooth speaker,   - 1 × WASP device (i.e. Bluetooth enhancer)   - selection of sports equipment (e.g. balls, cones). |
| Parrish, 2018 | - 5 stand-biased students desks - 2 free standing white board (with wheels) - 2 standing outdoor tables - Timers |
| Sudholz, 2016 | - Height-adjustable desks and large backless laboratory stools. - All traditional furniture removed |
| Torbeyns, 2017 | - Height adjustable bike desk (participants instructed to cycle for 4 class hours (4 x 50 min) per week. |
| Verloigne, 2018 | - Standing desks (3 per classroom, to avoid school barrier of high cost), - No traditional desk were removed. - Range students per classrrom (13-27) |
| Yang, 2017 | - Materials painted in school wall encouraging PA (climbing school stairs and walk in hallway) |
| **Incentives/rewards:** incentives to promote or reward physical activities or certain goals | |
| Ahmed et al., 2022 | - The participating students received a certificate (as an incentive) at the end of the intervention for their participation. |
| Bell, 2017 | - Acknowledgement: - Certificate and £10 gift vouchers |
| Corder, 2016 | - Points accumulated for engaging in activities - competition for most points with prizes for reaching point levels |
| Corder, 2020 | - Activity points for activity participation in and outside of school irrespective of duration or intensity (log ‘activity points’ on the GoActive website to unlock rewards) |
| James, 2020 | - 4 x voucher ($20 pounds each month, in increments by $5). To spent on: on PA (e.g. gym membership/sports club, buy equipment) |
| Schofield, 2005 | - Incentive to attend meeting (movie tickets) |
| **Physical activity sessions:** Opportunities for PA (supervised or unsupervised) during schools’ hours (e.g. during recess and lunchbreak) | |
| Ahmed et al, 2022 | - The students were encouraged to participate in a supervised sports activity once a week for 20 min during lunchtime, using the sports equipment |
| Altunkurek, 2019 | - 90 min PA session 1 day/week (warm up, stretching, activities incl. basketball, volleyball, football) |
| Amoah et al, 2021 | - aerobic and anaerobic exercises were delivered by a physical education health instructor. - 25–30 minutes per session |
| Ardic, 2016 | - 10-15 min PA/session - Once a week/15 weeks |
| Barbosa Filho, 2016,  Barbosa Filho, 2017,  Barbosa Filho, 2019,  Bandeira, 2020 | - Opportunities for PA supervised 10-15 min (“Gym in School”), 2/week (at free-time). Included: physical (stretching, located exercises), dynamic (e.g., games and rhythmic activities). In small and large groups. - By staff member. - School's open spaces. |
| Bonhauser, 2005 | - Activity session: - Part 1: Activity with no weight transfer: stretching, and arm, leg and trunk movement. - Part 2: weight transfer activities: large muscle movements   (fast walking, running and jumping).   - Part 3: sports practice (Women chose dance, aerobics, track practice and volleyball. |
| Budde, 2010 | - 12-minute session of running on a 400m track at set HR intensities (Group 1 = 50-65% HR max, group 2=70-85% HR max) |
| Bush, 2010,  Laberge, 2012 | - 12 different popular and culturally appropriate activities offered during the first 45 mins of 75 min lunch break (up to 3 per day) 2-5 days per week (including the video game Dance Revolution, Cardio-Surprise, kung fu, capoeira, hip-hop, African dance, a Swiss ball activity (which we named Abdominator), and a multisport (basketball and soccer) World Cup. |
| Carlin, 2018 | - 10-15 minute walks spread across the school week before the first bell, mid-morning break and lunch time |
| Costigan, 2018 | - 3 HIIT sessions per week (2 at beginning of PE, 1 during lunchtime) - intervention participants allocated to either Aerobic Exercise or Resistance and Aerobic programs |
| Dewar, 2013,  Dewar, 2014,  Lubans, 2012a | - Enhanced school sport sessions - Lunch-time physical activity sessions |
| Haapala, 2017 | - School gym: muscular training - Regular physically active morning assemblies - Walks during the school day, - Whole-school events involving sports and PA - Lunch breaks physical activity led by students, and |
| Hollis, 2016,  Sutherland, 2016a  Sutherland, 2016b  Sutherland, 2020  Sutherland, 2021 | - PA school breaks (recess and lunch), activities and equipment > 2/week |
| Kennedy, 2019  Kennedy, 2018  Kennedy, 2021 | - Structured physical activity program, teacher delivered, involving the following: activities such as bodyweight and elastic tubing RT; high-intensity resistance-training fitness challenges; strength-, flexibility-, and aerobic-based activities; and modified ball games. - Lunchtime fitness sessions, student directed: Teachers were asked to facilitate a minimum of 5 lunchtime sessions over the 10-wk intervention period. |
| Kolle, 2020 | - a 30 min/week lesson of physically active learning where play-based activities were integrated into other curriculum subjects (i.e. math, English, Norwegian). The classroom teacher for the subject planned and taught the lesson. - Student lead “Be happy” lesson 60min/week). Students grouped by Hobbies (e.g., football or handball), lifestyle sports (e.g., parkour or BMX cycling), dancing, and outdoor recreation. |
| Lazorick, 2015 | - Schedule daily 25 min supervised PA period outside or in wellness center |
| Leme, 2016,  Leme, 2018 | - School break PA sessions (14 x 15 minutes) |
| Lubans, 2010 | - Progressive free weight resistance training program delivered delivered during lunchtime twice a week - Progressive elastic tubing resistance training program delivered delivered during lunchtime twice a week |
| Lubans, 2011,  Lubans, 2012b,  Morgan, 2012 | - Enhance school sport (resistance training) - Enhanced school sport sessions (info and PA) - 10 x 90mins - Lunchtime activity sessions - 8 x 30min |
| Lubans, 2016b,  Lubans, 2016a,  Smith, 2014 | - Enhanced school sport sessions, 2 x 90min/session by school teachers. With elastic tubing resistance training aerobic/strength activities, fitness challenges, ball games and cool down (behavioral messages) |
| Ludyga, 2018 | - 20 min, 5/week, 8 weeks structured exercise, 5-10 min after lunch, supervised, by 2 instructors mix of aerobic and coordinative exercises: relay games, ball games, playing tag (cognitive challenging) |
| Ludyga, 2019 | - Intervention 1= moderate intensity (ratio 30s exercise, 30s recovery), 4 min warm-up/16 min activity), supervised by instructor as circuit training. Include: jumping jacks, 10 m shuttle run, rope skipping, stepping up down on a bench, jumping sideways, dribbling a ball while running. - Intervention 1= high intensity (ratio 60s exercise, 30s recovery) 4 min warm-up/16 min activity) supervised by instructor as circuit training. Include: jumping jacks, 10 m shuttle run, rope skipping, stepping up down on a bench, jumping sideways, dribbling a ball while running |
| Murphy et al, 2022 | - The activities offered were camogie (an indigenous sport to women in Ireland similar to field hockey), football, soccer, tennis, volleyball and badminton. Two activities were offered at each lunchtime and participants could select whichever one they preferred. Lunchtime games were based on small-group activities of —four to six participants per group to allow for connections to develop between participants |
| Mavilidi, 2020,  Lubans, 2021 | - HIT strategies:   - Gym-HIIT—combination of aerobic (e.g. skipping) and strength exercises (e.g. squat jumps),   - Sport-HIIT—using sports equipment (e.g. shuttle run while dribbling a basketball),   - Class-HIIT—exercises that can be performed in a standard classroom (e.g. running on the spot, triceps dips)   - Quick-HIIT—using Tabata protocol (e.g. 20 s intense work, followed by 10 s rest).   - Length: 8 to 20 min   - HR max: 85% |
| Melnyk, 2013,  Melnyk, 2015 | - 15-20/session, e.g. walking, dancing, kick-boxing movements. - Pedometer use (re-inforce intervention): encourage to increase step count by 10% (add steps on tracking sheet) |
| Peralta, 2009 | - 2/20 min lunchtime PA session with eleventh Grade students peer facilitated (Attended one 20 min training session) |
| Subramanian, 2015 | - Structured PA (Based WHO guidelines) by PE teachers   - 60 m/day to meet 30 min/day   - 30 mid/day 3/week of VPA and muscle strengthening   - Intensity by effort scale or random pulse rate   - For non-athlete: 2 h/day, 6 d/week, 6 months   - Athlete: (+ usual sport activity): 2 h/day, 6 d/week, 6 months - Non-structured PA: supervised, non-structured. Free to choose any: indoor, gym, music, drawing, craft, outdoor a football, basketball, skipping, gardening, badminton, khokho.   - For non-athlete: 2 h/day, 6 d/week, 6 months   - Athlete: (+ usual sport activity): 2 h/day, 6 d/week, 6 months |
| Tarp, 2016 | - Weekly, by volunteer students and teachers (4 hours course for students and teachers) |
| Yli-Piipari, 2016 | - 15 min (warm-up, cardiovascular endurance, strength training, flexibility exercises) |
| **Peer support:** Adolescent leaders to encourage PA among their peers | |
| Aceves-Martins, 2017 | - Adolescent Challenge Creators (ACC) selected by teachers provided with 4 hr training on social marketing principles and healthy lifestyle theory. ACCs designed and implemented activities to engage school peers (1.3 hrs/week for 24 weeks) to increase fruit and vegetables consumption, increase MVPA and/or reduce screen-time |
| Bell, 2017 | - Peer nomination: questionnaire to nominate an influential peer. - Peer support/recruitment: meet nominees, invite to training, peer supporters receive a diary to record relevant interactions with their peers. Diaries include:   - additional information and some ‘healthy challenges’ to encourage achievable changes in the peer supporters’ behaviour |
| Bogart, 2016 | - Peer leader club to increase student advocacy using role-plays and motivational interviewing to promote PA and health eating |
| Carlin, 2018 | - Structured peer-led (15-17 year olds) 10-15 minute walks spread across the school week before the first bell, mid-morning break and lunch time |
| Corder, 2016 | - Two weekly activities in tutor groups designed to use little or no equipment and different from typical school sports   - Mentors (older adolescents) and in-class peer leaders encourage participation in activities. |
| Corder, 2020 | - Year 9 tutor group (class or homeroom) chose 2 activities each week from a selection provided. - GoActive targeted peer-led class-based activity, with participation also encouraged outside of school. Older adolescent mentors encouraged Year 9 students to try at least 1 weekly GoActive session. - First 6 weeks, by intervention facilitators (by local councils) |
| Cui, 2012 | - Peer leader recruitment and training (three after-school 90 min workshops) - Student action (students encouraged to maintain healthy lifestyle based on personal goals set in the lesson), peer leaders encouraged to be role models and help facilitate other students maintenance of healthy lifestyles |
| Ghammam, 2017 | - Student leader groups organised an awareness day |
| Harrington, 2018  Gorely, 2019 | - Girls’ leadership: to influence PE, sport and PA in their schools/other girls. Role models, promote PA, market PA and received training. Teacher-student collaboration. |
| James, 2020 | - 10 /school: Peer mentors identify by students (peer nomination questionnaire). To support and encourage voucher use. - Workshop for training (by local council and support worker). Peer mentor purpose, develop on mentoring skills, then 1 every half term. |
| Lubans, 2012b,  Lubans 2011,  Morgan, 2012 | - Peer-training: (6 x 30 min) encourage to become PA leaders at school and home, with accreditation |
| Lubans, 2016b,  Lubans 2016a,  Smith, 2014 | - Student mentoring session- PA lunchtime, 6 x 20min/session   - lunch time mentoring session   - recruitment/training of grade 7 in elastic tubing resistance training |
| Sebire, 2018,  Sebire, 2019 | - Peer-supporter nomination: Who do you respect? Who are good leaders in sport or other group activities? Who do you trust? and Who do you look up to?).   - Girls with more nominations were invited. |
| Tymms, 2016 | - Peer mentoring: Linked PE classes in years 7 and 9 (other periods were used), 6/week lessons.   - In a classroom/gym or other suitable space. 1:1 (grade 9 are mentors).   - Mentoring sessions: 20–30 min/week for 6-weeks, intervention booklet (learning tasks), set PA goals, fill activity blog   - Teacher: overall class supervision.   - Weekly training session by IV teacher to grade 9 mentors: PA, behavioural techniques used and the skills/confidence to act as a mentor. |
| Van Woudenberg, 2018 | - Most influential adolescents, by peer nominations of classmates, each classroom.   - Trained to promote PA among their classmates.   - Training: 1. Introduction, 2. knowledge, 3. skills and, acceptance of the task. |
| **Research support:** Direct support from the research team involved | |
| Hollis, 2016,  Sutherland, 2016a  Sutherland, 2016b  Sutherland, 2020  Sutherland, 2021 | - School change agent: in school 1 day/week. Prompts: weekly email from change agent |
| James, 2020 | - Support worker engagement; 1/month: Attend school to increase students awareness, encourage of new activities. |
| Kennedy, 2018, Kennedy, 2021 | - Physical activity session observation and feedback (using the SAAFE teaching principles), provided by the research team   - Two Resistance Training for Teens sessions at each intervention school were observed by members of the research team using a structured SAAFE observation checklist. The checklist was used to assess intervention fidelity and provide feedback to teachers. Fidelity was assessed as compliance with the proposed session structure |
| Okely, 2017 | - Research support. Collect and interpret school data, assist with development and implementation of school action plan. Monthly meeting. Funds for implementation and TPD from Department of education: 2 days/training and 2 day/research symposium (mid-intervention) |
| **School policy:** Changes, adaptation of the school policies to encourage physical activity and reduce sitting time | |
| Hollis, 2016,  Sutherland, 2016a  Sutherland, 2016b  Sutherland, 2020  Sutherland, 2021 | - School PA policies: modified or under development (not commenced), partnership signed, and school committee established |
| Okely, 2017 | - Action plan, by each school committee to make intervention sustainable. Include:   - Lunch time and after school activities.   - Modified school policies (equipment and facilities use).   - Use of sport uniforms to school on sport days. |
| **Teacher training:** Teacher development sessions, pedagogical strategies. | |
| Barbosa Filho, 2016,  Barbosa Filho, 2017,  Barbosa Filho, 2019,  Bandeira, 2020 | - Teacher training;   - All intervention teachers,   - 4/hours:   - Health, school and academic performance.   - Received a manual.   - Teachers were encouraged to conduct lessons on health issues from manual or create/ implement similar strategies. - PE teacher session:   - 4/hours   - Received a manual   - Also material for Health-related teachers.   - Students produced poster and text with health issues. |
| Chen, 2020 | - The teachers received training through preparatory webinars on how to administer the questionnaire. |
| Contardo Ayala, 2018,  Sudholz, 2020 | - Teacher development session: A one-hour professional development session was held for the teachers timetabled to use the intervention classroom. This session included a presentation (1.5 hrs) by the research team outlining the study purpose and supportive prompts (outlined in detail below). Teachers were provided with the printed manual used for this information session. |
| Harrington, 2018  Gorely, 2019 | - Teacher development session: 1. write school self-review, 2. attend TPD (training day) by YST tutor (organisation Youth Sport Trust, developed the IV), how to establish peer leader group, do school action plan. |
| Hollis, 2016,  Sutherland, 2016a  Sutherland, 2016b  Sutherland, 2020  Sutherland, 2021 | - Teacher development session, 1 PE/school, 2 hour session |
| Kariippanon, 2019 | - The teaching approach in the flexible learning spaces was student-centered and group-work focused. Students were also afforded considerable freedom to choose how to go about their learning. - Together with the furniture available, this teaching approach created opportunities and incentives for students to move throughout the lesson |
| Kennedy, 2018  Kennedy, 2021 | - Accredited teacher training workshop: Two male and 2 female teachers from each intervention school were invited to attend the 1-d professional development workshop, which addressed all aspects of Resistance Training for Teens intervention. This included the following: 1) teacher roles and expectations, 2) intervention components, 3) introduction to RT and safety implications, and 4) philosophy |
| Knebel, 2020 | - For PE: 4 hr meeting, health outcomes, activities to increase enjoyment, and engaging a broader number of students in PE classes *handbooks (physical activity; life and health; sports; body and rhythmic practices). - General teachers: 4 hrs training session. Info about: guidelines for screen time, consequences of intensified exposure to electronic media on adolescents' health. Handbook, which covers activities related to health and screen time use adapted to the context of each of the school subjects. |
| Lazorick, 2015 | - Teacher development session: Teacher training and lesson plans This is the teachers' training that occurred two weeks prior the intervention. Do we need to include this as a strategy? |
| Lubans, 2016b,  Lubans 2016a,  Smith, 2014 | - Teacher development session: 2 6/hours workshop (pre and mid intervention), rationale, strategies and theory of the intervention - Fitness instructor session: 1 visit during sport session, teacher observe and fill observation check list. |
| Mavilidi, 2020 | - Teacher development session: training, resources and support, 6 hrs:   - Teaching strategies: to facilitate the delivery of at least two HIT activity breaks per week during curriculum time   - With Evidence VPA on cognitive, mental health - Practical sections: Creation of action plans. Aim: 2 least two HIT training sessions/week during lesson time. - Teacher workshop: 1-day professional learning workshop delivered by the research team |
| Melnyk, 2013 | - Teacher development session: 1 full/day, for health teachers. Teacher deliver the 15 sessions |
| Parrish, 2018 | - Teacher development session - before the study with additional support during IV by email/phone or school visits |
| Tymms, 2016 | - Teacher development session: Intervention manual, 2/hrs session |
| Verloigne, 2018 | - Teacher development session: Presentation PPT for pedagogical strategy, 10 min, to the teachers, health context of the intervention:   - SB definition,   - Consequences of too much sitting,   - Strategies to reduce school related sitting   - Info about the desks.   - Teacher asked: use a rotation system, students rotated every 25 mins from tradition to standing desks.   - Poster prompt to motive: Standing at your desk every day, keeps the doctor away: |
| **Technology:** Use of. text messages, apps, websites, etc as an intervention strategy or as an implementation strategy. | |
| Aceves-Martins, 2017 | - Use of social media (Facebook and Instagram) for dissemination |
| Corepal, 2019 | - StepSmart Challenge website |
| Haerens, 2006,  Haerens, 2007 | - Computer-tailored intervention: got tailored feedback about their intentions, attitudes, self-efficacy, social support, knowledge, benefits and barriers related to physical activity. |
| Kennedy, 2018  Kennedy, 2021 | - Web-based smartphone app: 1) an exercise library (of predominantly resistance-training exercises) with GIF animated images and descriptions of exercises, 2) a list of 7-min high-intensity resistance-training workouts of varying intensities with built-in countdown timer and results entry option, 3) the RTSB checklist for evaluating and improving RT movement skill competency, 4) tailored motivational messaging via twice-weekly e-mails to reinforce the 5 behavioral messages, 5) self-monitoring function for recording and reviewing physical activity, and 6) goal setting to promote participation in MVPA. Sex targeting strategies: Separate smartphone apps for boys and girls; however, both have the same functionality. The only differences between the apps are the appearance, with images of a same-sex role model and different color schemes. |
| Lubans, 2016b,  Lubans 2016a,  Smith, 2014 | - Smartphone app, Website, 15 weeks   - PA monitoring   - fitness challenged results   - motivational messages   - peer assessment of RT skills   - goal setting |
| Mavilidi, 2020 | - Burn to learn app: (i) description of a variety of different activity breaks (e.g. gym, sport, etc.); (ii) options for ‘solo’, ‘group’ or ‘class’ sessions utilising Bluetooth heart rate monitoring; (iii) timer, audible prompts and display of heart rate during activity breaks; (iv) personalised post session reports outlining heart rate average and peak heart rate (both at individual and whole class level); (v) display of session log on app dashboard. Students’ participation in the activity breaks were tracked using the B2L app and via teacher-reported session logs. |
| Suchert, 2015 | - document their steps and experiences using an interactive user account on the project homepage |
| Tarp, 2016 | - Activity watch: sum up time in PA during class time |
|  |  |

**Supplementary Table 3 Summary of the intervention strategies used (n) and their reported impact on different outcomes**

|  |  | **Intervention strategies** | | | | | | | | | | |
| --- | --- | --- | --- | --- | --- | --- | --- | --- | --- | --- | --- | --- |
| **Outcomes** | **Overall**  **(n of articles)** | **Active lesson**  **(n)** | **Community**  **Involvement**  **(n)** | **Educational**  **(n)** | **Environment**  **(n)** | **Incentives**  **(n)** | **Peer support**  **(n)** | **PA session**  **(n)** | **Research**  **Support**  **(n)** | **School policy**  **(n)** | **Teacher**  **Training**  **(n)** | **Technology**  **(n)** |
| **PA** |  |  |  |  |  |  |  |  |  |  |  |  |
| Decrease | **10** | 0 | 5 | 6 | 3 | 3 | 6 | 2 | 1 | 1 | 5 | 1 |
| Null | **16** | 5 | 9 | 10 | 2 | 10 | 6 | 1 | 1 | 0 | 1 | 2 |
| Increase | **24** | 1 | 4 | 13 | 14 | 3 | 9 | 11 | 2 | 2 | 12 | 4 |
| **SED** |  |  |  |  |  |  |  |  |  |  |  |  |
| Decrease | **13** | 1 | 3 | 7 | 9 | 1 | 7 | 8 | 0 | 0 | 6 | 4 |
| Null | **7** | 0 | 0 | 6 | 5 | 0 | 2 | 2 | 0 | 0 | 3 | 3 |
| Increase | **11** | 2 | 3 | 5 | 4 | 2 | 5 | 4 | 1 | 1 | 1 | 0 |
| **EE** |  |  |  |  |  |  |  |  |  |  |  |  |
| Decrease | **0** |  |  |  |  |  |  |  |  |  |  |  |
| Null | **0** | 0 | 0 | 0 | 0 | 0 | 0 | 0 | 0 | 0 | 0 | 0 |
| Increase | **2** | 1 | 0 | 0 | 2 | 0 | 0 | 0 | 0 | 0 | 1 | 0 |
| **Academic Outcomes** |  |  |  |  |  |  |  |  |  |  |  |  |
| Decrease | **0** |  |  |  |  |  |  |  |  |  |  |  |
| Null | **3** | 1 | 0 | 2 | 2 | 0 | 0 | 3 | 0 | 0 | 1 | 3 |
| Increase | 9 | 1 | 0 | 2 | 3 | 0 | 2 | 7 | 1 | 0 | 4 | 1 |
| **BMI** |  |  |  |  |  |  |  |  |  |  |  |  |
| Decrease | **11** | 1 | 1 | 7 | 4 | 0 | 6 | 9 | 1 | 1 | 4 | 1 |
| Null | **11** | 0 | 2 | 8 | 5 | 1 | 5 | 9 | 0 | 0 | 3 | 2 |
| Increase | **1** | 1 | 0 | 0 | 1 | 0 | 0 | 0 | 0 | 0 | 1 | 0 |
| **WC** |  |  |  |  |  |  |  |  |  |  |  |  |
| Decrease | **2** | 2 | 0 | 0 | 1 | 0 | 0 | 0 | 0 | 0 | 1 | 0 |
| Null | **6** | 1 | 1 | 5 | 3 | 0 | 3 | 6 | 0 | 0 | 1 | 2 |
| Increase | **0** | 0 | 0 | 0 | 0 | 0 | 0 | 0 | 0 | 0 | 0 | 0 |
| **Body fat** |  |  |  |  |  |  |  |  |  |  |  |  |
| Decrease | **5** | 0 | 1 | 4 | 4 | 0 | 3 | 4 | 0 | 0 | 0 | 0 |
| Null | **3** | 0 | 1 | 1 | 2 | 0 | 1 | 3 | 0 | 0 | 1 | 2 |
| Increase | **0** | 0 | 0 | 0 | 0 | 0 | 0 | 0 | 0 | 0 | 0 | 0 |
| **Blood pressure** |  |  |  |  |  |  |  |  |  |  |  |  |
| Decrease | **4** | 1 | 0 | 2 | 1 | 1 | 1 | 2 | 1 | 0 | 0 | 0 |
| Null | **1** | 0 | 0 | 0 | 0 | 0 | 1 | 1 | 0 | 0 | 0 | 0 |
| Increase | **0** | 0 | 0 | 0 | 0 | 0 | 0 | 0 | 0 | 0 | 0 | 0 |
| **CRF** |  |  |  |  |  |  |  |  |  |  |  |  |
| Decrease | **0** |  |  |  |  |  |  |  |  |  |  |  |
| Null | **4** | 0 | 0 | 2 | 0 | 0 | 1 | 3 | 0 | 0 | 0 | 1 |
| Increase | **6** | 2 | 0 | 1 | 2 | 1 | 1 | 4 | 1 | 0 | 1 | 2 |
| **Muscle fitness** |  |  |  |  |  |  |  |  |  |  |  |  |
| Decrease | **0** |  |  |  |  |  |  |  |  |  |  |  |
| Null | **5** | 1 | 0 | 1 | 1 | 0 | 0 | 5 | 0 | 0 | 0 | 1 |
| Increase | 5 | 0 | 2 | 3 | 4 | 0 | 2 | 4 | 0 | 0 | 2 | 2 |
| **Wellbeing** |  |  |  |  |  |  |  |  |  |  |  |  |
| Decrease | **1** | 0 | 0 | 0 | 1 | 0 | 0 | 0 | 0 | 0 | 1 | 0 |
| Null | **2** | 0 | 0 | 0 | 1 | 1 | 1 | 1 | 0 | 0 | 1 | 1 |
| Increase | 9 | 0 | 1 | 4 | 2 | 1 | 5 | 8 | 0 | 0 | 2 | 1 |
| **Academic Outcomes** |  |  |  |  |  |  |  |  |  |  |  |  |
| Decrease | **0** |  |  |  |  |  |  |  |  |  |  |  |
| Null | **3** | 1 | 0 | 2 | 2 | 0 | 0 | 3 | 0 | 0 | 1 | 3 |
| Increase | 12 | 3 | 0 | 2 | 3 | 0 | 2 | 9 | 1 | 0 | 4 | 1 |

Abbreviations: n=number; PA= physical activity; SED= sedentary behaviour; EE= energy expenditure, BMI, body mass index; WC=waist circumference; CRF=cardiorespiratory fitness. Decrease=decrease effect on the outcome; Null: no effect on the outcomes; Increase: increase effect on the outcome.

**Supplementary Table 4 Methodological quality assessment**

## Supplementary Table 5: PRISMA 2020 checklist

| **Section and Topic** | **Item #** | **Checklist item** | **Location where item is reported** |
| --- | --- | --- | --- |
| **TITLE** | | |  |
| Title | 1 | Identify the report as a systematic review. | 1 |
| **ABSTRACT** | | |  |
| Abstract | 2 | See the PRISMA 2020 for Abstracts checklist. | 2 |
| **INTRODUCTION** | | |  |
| Rationale | 3 | Describe the rationale for the review in the context of existing knowledge. | 4 |
| Objectives | 4 | Provide an explicit statement of the objective(s) or question(s) the review addresses. | 5 |
| **METHODS** | | |  |
| Eligibility criteria | 5 | Specify the inclusion and exclusion criteria for the review and how studies were grouped for the syntheses. | 6 |
| Information sources | 6 | Specify all databases, registers, websites, organisations, reference lists and other sources searched or consulted to identify studies. Specify the date when each source was last searched or consulted. | 7 |
| Search strategy | 7 | Present the full search strategies for all databases, registers and websites, including any filters and limits used. | Supplementary Table 1 |
| Selection process | 8 | Specify the methods used to decide whether a study met the inclusion criteria of the review, including how many reviewers screened each record and each report retrieved, whether they worked independently, and if applicable, details of automation tools used in the process. | 7 |
| Data collection process | 9 | Specify the methods used to collect data from reports, including how many reviewers collected data from each report, whether they worked independently, any processes for obtaining or confirming data from study investigators, and if applicable, details of automation tools used in the process. | 8 |
| Data items | 10a | List and define all outcomes for which data were sought. Specify whether all results that were compatible with each outcome domain in each study were sought (e.g. for all measures, time points, analyses), and if not, the methods used to decide which results to collect. | 8 |
|  | 10b | List and define all other variables for which data were sought (e.g. participant and intervention characteristics, funding sources). Describe any assumptions made about any missing or unclear information. | 8 |
| Study risk of bias assessment | 11 | Specify the methods used to assess risk of bias in the included studies, including details of the tool(s) used, how many reviewers assessed each study and whether they worked independently, and if applicable, details of automation tools used in the process. | 9 |
| Effect measures | 12 | Specify for each outcome the effect measure(s) (e.g. risk ratio, mean difference) used in the synthesis or presentation of results. | 10 |
| Synthesis methods | 13a | Describe the processes used to decide which studies were eligible for each synthesis (e.g. tabulating the study intervention characteristics and comparing against the planned groups for each synthesis (item #5)). | 9-10 |
|  | 13b | Describe any methods required to prepare the data for presentation or synthesis, such as handling of missing summary statistics, or data conversions. | 9-10 |
|  | 13c | Describe any methods used to tabulate or visually display results of individual studies and syntheses. | 9-10 |
|  | 13d | Describe any methods used to synthesize results and provide a rationale for the choice(s). If meta-analysis was performed, describe the model(s), method(s) to identify the presence and extent of statistical heterogeneity, and software package(s) used. | 10-11 |
|  | 13e | Describe any methods used to explore possible causes of heterogeneity among study results (e.g. subgroup analysis, meta-regression). | 10-11 |
|  | 13f | Describe any sensitivity analyses conducted to assess robustness of the synthesized results. | N/A |
| Reporting bias assessment | 14 | Describe any methods used to assess risk of bias due to missing results in a synthesis (arising from reporting biases). | 10 |
| Certainty assessment | 15 | Describe any methods used to assess certainty (or confidence) in the body of evidence for an outcome. | 9-10 |
| **RESULTS** | | |  |
| Study selection | 16a | Describe the results of the search and selection process, from the number of records identified in the search to the number of studies included in the review, ideally using a flow diagram. | 10-11 |
|  | 16b | Cite studies that might appear to meet the inclusion criteria, but which were excluded, and explain why they were excluded. | Figure 1 |
| Study characteristics | 17 | Cite each included study and present its characteristics. | 11-12, Table 1 |
| Risk of bias in studies | 18 | Present assessments of risk of bias for each included study. | 16 Supplementary Material |
| Results of individual studies | 19 | For all outcomes, present, for each study: (a) summary statistics for each group (where appropriate) and (b) an effect estimate and its precision (e.g. confidence/credible interval), ideally using structured tables or plots. | Figure 2 - 5 |
| Results of syntheses | 20a | For each synthesis, briefly summarise the characteristics and risk of bias among contributing studies. | 10-16 |
|  | 20b | Present results of all statistical syntheses conducted. If meta-analysis was done, present for each the summary estimate and its precision (e.g. confidence/credible interval) and measures of statistical heterogeneity. If comparing groups, describe the direction of the effect. | 10-16 |
|  | 20c | Present results of all investigations of possible causes of heterogeneity among study results. | 13-14, 18-19 |
|  | 20d | Present results of all sensitivity analyses conducted to assess the robustness of the synthesized results. | N/A |
| Reporting biases | 21 | Present assessments of risk of bias due to missing results (arising from reporting biases) for each synthesis assessed. | 16-16 |
| Certainty of evidence | 22 | Present assessments of certainty (or confidence) in the body of evidence for each outcome assessed. | 13-14, 18-19 |
| **DISCUSSION** | | |  |
| Discussion | 23a | Provide a general interpretation of the results in the context of other evidence. | 16-17 |
|  | 23b | Discuss any limitations of the evidence included in the review. | 19 |
|  | 23c | Discuss any limitations of the review processes used. | 19 |
|  | 23d | Discuss implications of the results for practice, policy, and future research. | 19 |
| **OTHER INFORMATION** | | |  |
| Registration and protocol | 24a | Provide registration information for the review, including register name and registration number, or state that the review was not registered. | 6 |
|  | 24b | Indicate where the review protocol can be accessed, or state that a protocol was not prepared. | 6 |
|  | 24c | Describe and explain any amendments to information provided at registration or in the protocol. | N/A |
| Support | 25 | Describe sources of financial or non-financial support for the review, and the role of the funders or sponsors in the review. | 20 |
| Competing interests | 26 | Declare any competing interests of review authors. | 20 |
| Availability of data, code and other materials | 27 | Report which of the following are publicly available and where they can be found: template data collection forms; data extracted from included studies; data used for all analyses; analytic code; any other materials used in the review. | N/A |

*From:*  Page MJ, McKenzie JE, Bossuyt PM, Boutron I, Hoffmann TC, Mulrow CD, et al. The PRISMA 2020 statement: an updated guideline for reporting systematic reviews. BMJ 2021;372:n71. doi: 10.1136/bmj.n71

For more information, visit: <http://www.prisma-statement.org/>

## Supplementary 6: Publication Bias – Funnel plots

**
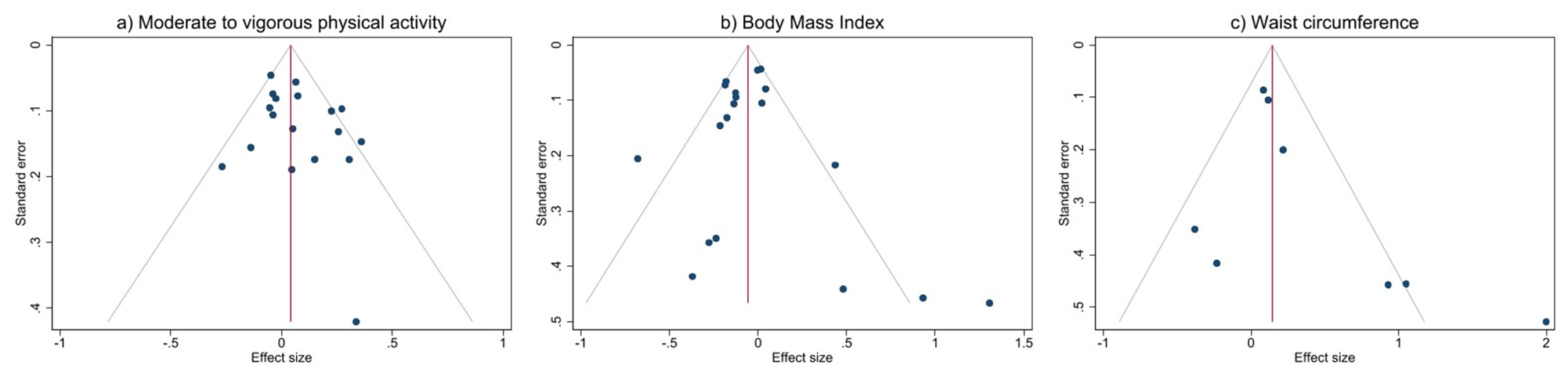
**

Funnel plots of standard error by effect size of effect of school-based initiatives on a) Moderate to vigorous physical activity, b) body mass index, c) waist circumference.

## Supplementary 7: Summary statistics used for calculation of standardized mean difference across the studies.

| **Study** | **Result (95% CI)*** | **n** | **Calculated  effect size** | **SE** |
| --- | --- | --- | --- | --- |
| **SED** |  |  |  |  |
| Andrade et al. 2014 [70] | -18.1(-50.8,-14.6) | IV=550, CG=684 | -0.2502 | 0.173 |
| Bell et al. 2017 [42] | 14.77 (4.05,25.49) | IV=310, CG=304 | 0.276 | 0.102 |
| Corder et al. 2020 [73] | 5.34 (-7.6, 18.3) | IV=1,414, CG=1,224 | 0.0375 | 0.046 |
| Dewar et al. 2014 [103] | 0.56 (-2.84, 3.97) | IV=108, CG=113 | 0.0557 | 0.155 |
| Haapala et al. 2017 [86] | 0.53 (-0.43, 1.49) | IV=125, CG=93 | 0.2201 | 0.205 |
| Harrington et al. 2018 [36] | -2.64 (-13.03, 7.75) | IV=867, CG=885 | -0.0281 | 0.056 |
| Okely et al. 2017 [38] | -2.81 (-21.74, 16.19) | IV=566, CG=633 | -0.0236 | 0.081 |
| Sebire et al, 2018 [39] | -31.8 (-57.44, -6.18) | IV=225, CG=128 | -0.3093 | 0.128 |
| **LPA** |  |  |  |  |
| Andrade et al. 2014 [70] | 4.6 (-14.6, 23.8) | IV=550, CG=684 | 0.1725 | 0.173 |
| Corder et al. 2020 [73] | -9.46 (-23.35, 4.44) | IV=1,414, CG=1,224 | -0.0618 | 0.046 |
| Haerens et al. 2007 [98] | 27.5 (-12.01, 67.01) | IV=1,414, CG=1,224 | 0.2359 | 0.174 |
| Harrington et al, 2018 [36] | 1.5 (-5.93, 8.94) | IV=843, CG=714 | 0.0223 | 0.056 |
| Okely et al. 2017 [38] | -4.69 (-17.1, 7.73) | IV=566, CG=633 | -0.0537 | 0.081 |
| **MPA** |  |  |  |  |
| Costigan et al, 2018 [46] | 0.53 (-5.18, 6.24) | IV=29, CG=18 | 0.0559 | 0.3 |
| Dewar et ak, 2014 [103] | -0.61 (-1.71,0.49) | IV=31, CG=57 | -0.1831 | 0.156 |
| Lubans et al. 2021 [99] | 6.64 (−0.50, 13.78) | IV=153, CG=182 | 0.0749 | 0.077 |
| Okely et al. 2017 [38] | -0.53 (-5.91, 4.85) | IV=566, CG=633 | -0.0165 | 0.081 |
| Peralta et al. 2009 [33] | 3.8 (-34.8, 42.4) | IV=245=191 | 0.0853 | 0.418 |
| Sutherland et al, 2016 [102 | 4.5 (2, 7) | IV=12, CG=11 | 0.3001 | 0.097 |
| **MVPA** |  |  |  |  |
| Andrade et al. 2014 [70] | 13.6 (-4.1, 30.8) | IV=550, CG=684 | 0.3048 | 0.174 |
| Bell et al. 2017 [42] | -0.41 (-1.82, 1) | IV=310, CG=304 | -0.0542 | 0.095 |
| Corder et al. 2016 [51] | 5.1 (1.1, 9.2) | IV=152; CG=68 | 0.3614 | 0.147 |
| Corder et al. 2020 [73] | -1.91 (-5.53, 1.7) | IV=1,414, CG=1,224 | -0.048 | 0.046 |
| Dewar et al. 2014 [103] | -0.62 (-2.08, 0.84) | IV=108, CG=113 | -0.1396 | 0.155 |
| Haerens et al. 2007 [35] | 4.8 (-6.4, 16) | IV=50; CG=67 | 0.1509 | 0.174 |
| Harrington et al. 2018 [36] | 1.67 (-1.15, 4.48) | IV=867, CG=885 | 0.0654 | 0.056 |
| Kennedy et al. 2018 [77] | -4.1 (-9.6, 1.4) | IV=61, CG=57 | -0.269 | 0.185 |
| Kolle et al. 2020 [65] | -4.1 (-8.1, -0.04) | IV=546; CG=288 | 0.0374 | 0.089 |
| Lubans et al. 2016 [108] | 0.1 (-0.8, 1) | IV=46; CG=71 | 0.0468 | 0.189 |
| Lubans et al. 2021 [99] | 6.08 (-1.25, 13.41) | IV=153, CG=182 | 0.0742 | 0.077 |
| Okely et al. 2017 [38] | -0.35 (-6.58, 5.87) | IV=566, CG=633 | -0.0248 | 0.081 |
| Peralta et al. 2009 [33] | 16.4 (-26.8, 59.6) | IV=245=191 | 0.3359 | 0.42 |
| Sebire et al. 2018 [39] | 1.11 (-4.31, 6.55) | IV=225, CG=128 | 0.051 | 0.127 |
| Sutherland et al. 2016 [102] | 7 (2.68, 11.4) | IV=12, CG=11 | 0.2723 | 0.097 |
| Tarp et al. 2016 [72] | 1.2 (3.9, 6.3) | IV=96; CG=148 | 0.2569 | 0.132 |
| Tymms et al. 2016 [58] | -2.5 (-11.84, 6.84) | IV=337; CG=392 | -0.039 | 0.074 |
| **BMI** |  |  |  |  |
| Amoah et al. 2021 [94] | -0.74 (-1.1, 0.3) | IV=419; 417 | -0.2781 | 0.357 |
| Andrade et al. 2014 [70] | -0.01 (-0.09, 0.08) | IV=550, CG=684 | -0.004 | 0.046 |
| Ardic et al. 2016 [89] | 1.32 (0.001, 0.85) | IV=45, CG=42 | 0.4339 | 0.217 |
| Corder et al. 2020 [73] | 0.01 (-0.07, 0.10) | IV=1,543; CG=1,319 | 0.0143 | 0.044 |
| Dewar et al. 2013 [103] | -0.12 (-0.43, 0.08) | IV=113, CG=121 | -0.1767 | 0.131 |
| Hollis et al. 2016 [60] | -0.08 (-0.31, -0.05) | IV=560, CG=390 | -0.1809 | 0.066 |
| Kennedy et al. 2018 [77] | -0.13 (-0.311, 0.05) | IV=279, cg=188 | -0.1264 | 0.094 |
| Leme et al. 2016 [107] | -0.07 (-0.50, 0.07) | IV=111, CG=83 | -0.215 | 0.146 |
| Lubans et a. 2011 [104] | -0.2 (-1.08, -0.27) | IV=50, CG=50 | -0.6785 | 0.206 |
| Lubans et al. 2010_B [93] | -0.2 (-0.98, 0.35) | IV=31, CG=12 | -0.3185 | 0.342 |
| Lubans et al. 2010_G [93] | 0 (-0.64, 0.64) | IV=26, CG=14 | 0 | 0.331 |
| Lubans et al. 2012 [118] | -0.08 (-0.34, 0.07) | IV=178, CG=179 | -0.1373 | 0.106 |
| Lubans et al. 2021 [99] | 0.02 (-0.11, 0.19) | IV=312, CG=323 | 0.0412 | 0.079 |
| Melnyk et al. 2013 [67] | -0.2 (-0.32, -0.04) | IV=358, CG=421 | -0.1856 | 0.072 |
| Peralta et al. 2009 [33] | -0.2 (-0.92, 0.44) | IV=16, CG=17 | -0.2377 | 0.35 |
| Smith et al. 2014 [106] | 0 (-0.18, 0.22) | IV=181, CG=180 | 0.0213 | 0.105 |
| Tarp et al. 2016 [72] | -0.1 (-0.29, 0.04) | IV=194, CG=438 | -0.1274 | 0.086 |
| **WC** |  |  |  |  |
| Lubans et al. 2010_B [93] | 3.05 (0.61, 5.48) | IV=31; CG=12 | 0.2905 | 0.341 |
| Lubans et al. 2010_G [93] | -1.05 (-4.17, 2.07) | IV=26; CG=14 | -0.1193 | 0.332 |
| Lubans et al. 2011 [104] | 0.8 (-0.6, 2.3) | IV=50, CG=50 | 0.2163 | 0.201 |
| Peralta et al. 2009 [33] | -1.7 (-4.7, 1.4) | IV=16, CG=17 | -0.3805 | 0.351 |
| Smith et al. 2014 [106] | 0.5 (-0.38, 1.38) | IV=181, CG=180 | 0.1162 | 0.105 |
| Tarp et al. 2016 [72] | 0.7 (-0.7, 2.1) | IV=194, CG=438 | 0.0845 | 0.086 |

Data used to calculate standardised effect sizes: Adjusted mean difference between groups with 95% CI; group n's

Abbreviations: n=number; Sedentary time, light intensity physical activity, moderate physical activity, vigorous physical activity, moderate-to-vigorous physical activity. BMI, body mass index; WC=waist circumference;
